# Exploring the Intention–Behavior Relationship in Flood Adaptation Using Longitudinal Data

**DOI:** 10.1111/risa.70261

**Published:** 2026-05-11

**Authors:** Tang T. Luu, Annegret H. Thieken, Toon Haer, Tran Thi Tuyen, Philip Bubeck

**Affiliations:** ^1^ Institute of Environmental Science and Geography, University of Potsdam Potsdam‐Golm Germany; ^2^ Vrije Universiteit Amsterdam Amsterdam the Netherlands; ^3^ Faculty of Social Sciences and Arts Saigon University Ho Chi Minh City Vietnam

**Keywords:** flood adaptation intention, flood adaptation behavior, intention‒behavior gap, longitudinal study, Vietnam

## Abstract

Insights into factors motivating individual adaptation are needed to reduce flood damage, especially in lower‐income countries. According to key behavioral theories, adaptation intention leads to behavior and is, therefore, often used as a proxy for implementation. Empirical studies, however, report substantial intention‒behavior gaps (i.e., the percentage of intended actions unrealized) and limited predictive power of models built on these theories. The literature suggests that intention strength, which captures levels of commitment, could better predict behavior but has not been tested in the flood risk domain. Moreover, several predictors might influence intention and actual behavior differently or might even influence behavior independently. Studying the intention‒behavior relationship could thus help increase the theories' predictive power. Investigating the intention‒behavior relationship is challenging due to the lack of longitudinal surveys that capture behavior over time. Here, we deploy intention strength in a two‐wave longitudinal survey among 401 randomly selected respondents from Vietnam (95% retention rate) to examine the intention‒behavior relationship in preparing devices and retrofitting homes against flooding. We find that > 90% of intended adaptive actions indicated by respondents in Wave 1 went unrealized within 6 months. Intention strength seems unimportant for preparing devices, whereas the strongest intention significantly correlates with higher implementation of retrofitting homes. Studies using intention as a proxy for behavior could, therefore, consider intention strength instead. We also find various factors significantly motivating behavior but not intention, including financial capacity, experiencing large floods regularly, and housing situation. Policymakers, therefore, should focus more on these factors when considering adaptation strategies. Future research in other settings could contextualize the adjustment to behavioral theories.

## 1 Introduction

Flooding is among the most destructive natural hazards worldwide (IFRC 2020). Twenty‐three percent of the world's population lives in areas exposed to floods with inundation depths > 0.15 m, and 89% of flood‐exposed people live in low‐ and middle‐income countries (Rentschler et al. 2022). In 2024 alone, floods caused 5883 deaths and $32.8 billion in damage (CRED 2025). Flood risk reduction is thus urgently needed, especially for vulnerable communities. Various studies have emphasized that individual adaptive behavior plays a crucial role in mitigating current and future risks (Bubeck, Botzen, Kreibich, et al. 2012; Hudson et al. 2020; Kuhlicke et al. 2020).

Behavioral theories such as the protection motivation theory (PMT) and the theory of planned behavior (TPB) are often used to study individual behavior in the natural hazard domain (Kuhlicke et al. 2023). These theories hypothesize adaptation intention as a behavioral precursor. Research on intentions is needed and insightful if deriving actual behaviors is impossible, for example, when implementation of a measure is still rare (Botzen and van den Bergh 2012). Intention has also been suggested as a proxy for actual behavior to avoid reverse feedback effects of previously implemented measures, that is, causing a reduction in risk perception, thus underestimating its effect on behavior (Bubeck, Botzen, and Aerts 2012; Weinstein et al. 1998).

However, studies from various domains (e.g., health and water governance) found that despite including intentions, there remains a substantial amount of unexplained variance in behavior models (Conner and Norman 2022; Duong 2022; Maartensson and Loi 2022; Perera et al. 2023; Weinstein and Rothman 2005). Likewise, the predictive power of behavioral theories used in the flood risk domain (FRD) is limited (Kellens et al. 2013; Kuhlicke et al. 2020), and the contribution of intention is uncertain. Osberghaus et al. (2025) and Noll (2023) include intention as a predictor of behavior, and both report weak or no correlation. In addition, they found that 40% and 75% of intended measures were not implemented, referred to as the intention‒behavior gap (IBG), while approximately 93.2% of new implementations were not preceded by intention. Bubeck et al. (2020) report IBGs of between 21% and 56%.

Ajzen (1991) argues that receiving new information could change an intention after its formation, thus causing IBG. The higher the commitment to perform the behavior, the less likely new information is to change the intention (Ajzen 1991). The correlation between intention and behavior is thus assumed to be higher if intention was expressed with greater confidence (Ajzen 1991). Using similar reasoning, Rhodes and Rebar (2017) separate the intention concept into two meanings: “decisional intention” (i.e., a binary decision to implement a measure or not) and “intention strength” (i.e., the level of commitment to execute that decision). They find that intention strength captures the effects of cognitive variables on behavior better and is a superior predictor of behavior to decisional intention in the physical activity domain. In the FRD, only van Valkengoed et al. (2024) operationalized the intention with a (four‐point) Likert scale, although the concept of intention strength is not explicitly mentioned. The three studies on IBGs (Bubeck et al. 2020; Noll 2023; Osberghaus et al. 2025), however, employ the concept as a decisional intention. The large IBGs reported could thus be because intention was assessed uniformly as a decision rather than a strength.

Despite the substantial IBGs, many studies investigate decisional intention and thereby deduce adaptive policies for implementation (Maidl and Buchecker 2015; Noll et al. 2022; Tasantab et al. 2022), thus assuming identical drivers for intentions and behaviors (Osberghaus et al. 2025). Osberghaus et al. (2025) argue that if the predictors of intention and behavior are similar in terms of effect sizes and significance levels, it might be helpful to derive some insights from the intention model for behavior. They test this assumption but with respect to sociodemographic variables and conclude that although intention might not serve as a good predictor, the intention model may be used as a proxy to assess the drivers of behavior.

Studying whether the same set of predictors explains both intention and behavior could also help elucidate the pathways of intention and behavior in behavioral theories, thus increasing the theories' predictive power. If a predictor influences intention and behavior differently (a so‐called incongruent predictor), it could have an independent effect on behavior that is not mediated through intention as currently hypothesized by the theories, thus causing the IBG. If the predictor is influential for intention but unimportant for behavior, policies targeting this predictor may be irrelevant for behavior change. Even worse, if the predictor increases intention but reduces behavior, an enhancement of this predictor could reduce behavior and increase the IBG. Investigating whether the same predictors can explain both intention and behavior is thus crucial.

A challenge associated with studying the relationship between intention and behavior is a lack of panel (i.e., longitudinal) studies. A panel study collects data on the same individuals multiple times (Neuman 2014), therefore better capturing the dynamics and thus providing insights into individual responses toward flooding (Bubeck et al. 2020). Although powerful, the use of panel designs in the FRD is limited (Hudson et al. 2020), as they are costly and more complicated (Neuman 2014). Moreover, one of the major challenges associated with panel studies is attrition bias, i.e., respondents dropping out of the survey nonrandomly, which biases statistical findings (Hudson et al. 2020).

Despite these challenges, more panel studies, which systematically investigate the relationship between intention and behavior using a broad range of predictors, are necessary to better understand the intention‒behavior relationship (Conner and Norman 2022; Hagger et al. 2022; Hassan et al. 2016). Our research thus aims to explore the IBG in the context of a lower‐income country, deploying the concept of intention strength in a two‐wave panel survey over 6 months. To this end, we seek answers to the following questions:

1. How large is the IBG?
2. How does intention strength relate to subsequent adaptation behavior?
3. What are shared versus incongruent predictors of intention strength and behavior?

The findings are useful for policymakers in flood‐prone areas to design adaptation policies. Our findings can also provide valuable insights to scholars seeking to improve behavioral theories or modelers in sociohydrology that merge empirical and risk modeling research.

## 2 Case Study, Data, and Methods

### 2.1 Research Area and the Panel Survey

**FIGURE 1:**
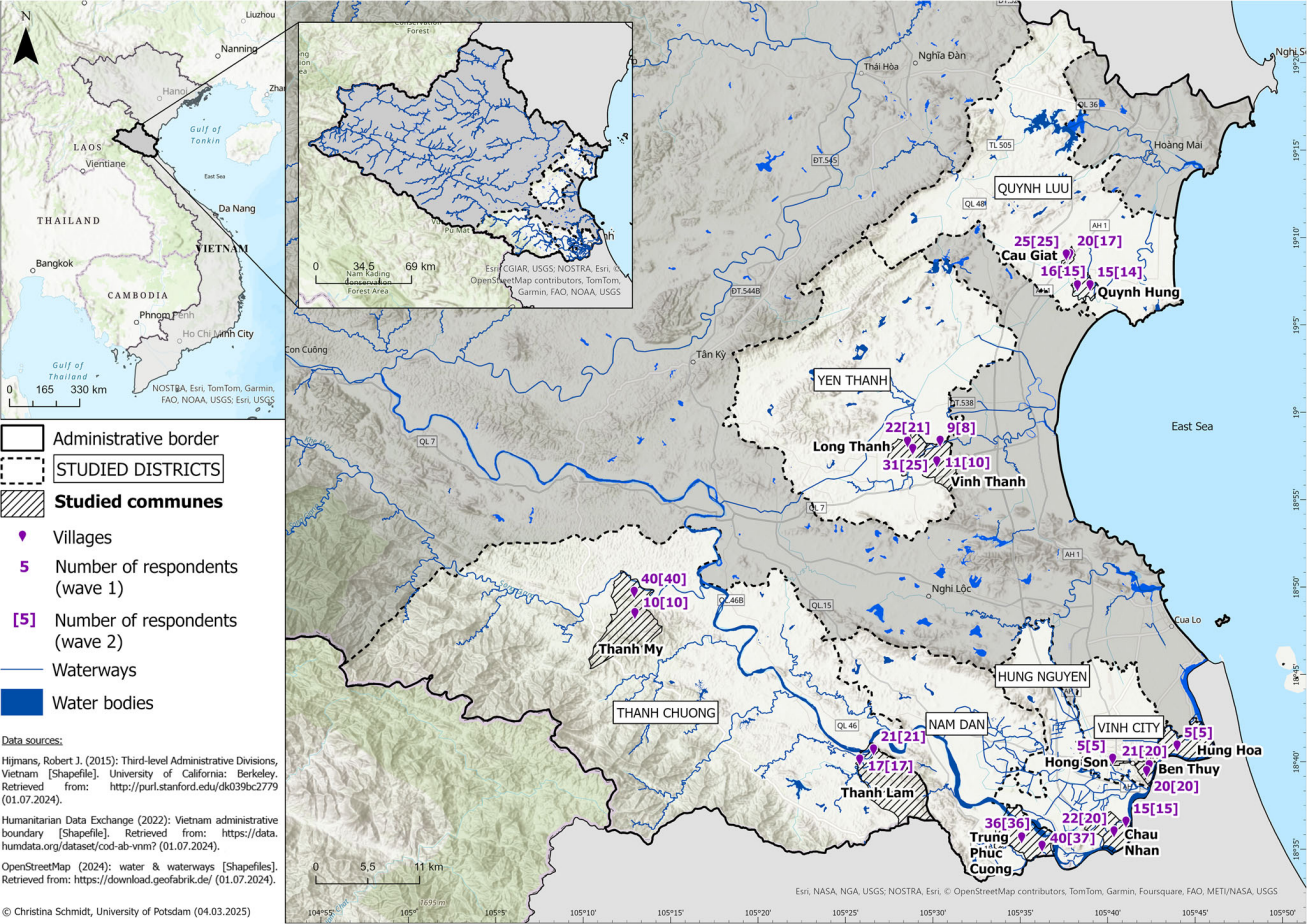
Case study areas (the areas are named as of 2023 and do not include the recent changes due to the merging of units in Vietnam, see https://en.wikipedia.org/wiki/Plan_to_arrange_and_merge_administrative_units_in_Vietnam_2024%E2%80%932025, retrieved on October 22 2025) of Nghe An Province. *Source*: Adjusted from T. T. Luu et al. (2025).

Vietnam is jointly ranked number one with Bangladesh in terms of exposure to flooding worldwide (WBG & ADB 2021). Among the country's provinces, Nghe An has the highest flood risk (C. Luu et al. 2019). Local people experience annual flooding in many areas of the province, with extreme floods occasionally causing severe damage. For example, flooding caused 166 deaths, inundated 9698 ha of rice, washed away 7235 houses, and broke or overflowed 382 dikes in 1978 (Nguyễn et al. 2016), 12 deaths and €49 million in damage in 2022 (NAPC 2023). In the aftermath of the 2022 flood, 20 villages from eleven communities in six districts were surveyed (Figure 1).

A pilot test of the questionnaire with 44 respondents was conducted in September 2023. A total of 401 respondents^1^ were then randomly selected for structured face‐to‐face interviews in Wave 1 during the flooding season in October 2023 by carefully trained local enumerators using KoboToolbox software (see details in T. T. Luu et al. 2025). The same respondents were contacted 6 months later during the dry season in April 2024. Although no flooding occurred between the two waves, there was a heat wave immediately before and during Wave 2. Different strategies were deployed to increase the retention rate, such as interviewing outside of working hours. Eventually, 381 respondents (a 95.01% retention rate^2^) were interviewed in Wave 2. The random selection and high retention rate in our study reduce possible issues of selection and attrition bias in longitudinal surveys (Hudson et al. 2020).

### 2.2 Variable Selection and Measurement

Two groups of adaptation behaviors, namely, preparing emergency devices and retrofitting homes, were studied. Preparing devices includes six specific measures: barrels (to store food), handmade rafts using barrels, wooden rafts, boats, life jackets, and shelves. Retrofitting homes includes seven specific measures: elevating the entrance, floor, or kitchen, replacing the floor/wall/roof with waterproof materials, preparing a garret inside the existing house, expanding the yard/garden, and building a flood‐proof hut beside the main house.

The outcome variable “behavior” was calculated by counting the number of specific measures implemented in each wave, separately for preparing devices and retrofitting homes. “Intention” was measured using a 6‐point Likert scale, ranging from *definitely no* (coded as 0) to *definitely will* (5), to capture intention strength (Ajzen 1991; Rhodes and Rebar 2017). Intention and behavior were treated as continuous in all models.

Predictors were selected based on the TPB and PMT^3^. These were categorized into individuals' perceived flood risks (i.e., probabilities and consequences), behavioral control (i.e., self‐efficacy and financial capacity, which are the perceived ability to act and cover financial costs associated with implementing the measure, respectively), response efficacy (i.e., the perceived effectiveness of the measure implemented) (Rogers 1983), and subjective norms (Ajzen 1991). Other predictors were selected from empirical studies: other social norms (descriptive and injunctive), personality, flood experience, maladaptation, housing situation, and sociodemographic factors (Bubeck et al. 2013; Grothmann and Reusswig 2006; Kuhlicke et al. 2020; T. T. Luu et al. 2025; Rhodes et al. 2022; van Valkengoed and Steg 2019). In total, 36 predictors were selected for preparing devices. The same predictors, except for newly built flood‐adapted houses, form the input predictors of retrofitting homes. A 6‐point Likert scale was used for cognitive variables where possible (see Appendix A for a complete list of variables and coding).

### 2.3 Data Processing

Certain measures implemented in the past are assumed to be maintained and thus recalled consistently over time. However, several respondents reported past implementation in Wave 1 but not in Wave 2 or reported different years of implementation between the two waves, thus causing inconsistencies. The inconsistent respondents are widely distributed among the sample (Appendix C). The lack of clustering in the inconsistencies suggests that it was probably due to incomplete recall (i.e., respondents forgot part of the measures they had taken in either Wave 1 or 2).

Inconsistencies in panel survey data are not uncommon in the FRD. We observed at least one case where inconsistent data were omitted (Osberghaus et al. 2025). This approach, however, might increase the retention bias (Hudson et al. 2020). Other panel surveys assume the continuity of past implementation, where implemented measures in previous waves were not asked again in later waves but would be added to behavior indexes (Bubeck et al. 2020; Noll 2023). We take a middle approach, adjusting for inconsistencies based on the assumption that past implementations should be maintained over time, except for barrels, life jackets, and wooden rafts, as these devices may be broken and discarded at some points (see Appendix C).

Before the adjustment, the average values of the behavior indexes for both measure groups decreased over time. After the adjustment, the direction for preparing devices is unchanged, although the slope is less steep; in contrast, it slightly increases for retrofitting homes (Figure C2, Appendix C). Since other explanations for the inconsistencies were considered (Appendix C), we believe the adjustment is justified. The resultant upward trend of retrofitting homes is arguably consistent with reality because the living standards in Vietnam are rising. Several other variables^4^ were also adjusted for inconsistencies based on the same reasoning.

### 2.4 Statistical Approach

#### 2.4.1 Quantifying the IBG

Respondents were first classified into the Yes‐spectrum and No‐spectrum based on their intention strength in Wave 1: the Yes‐spectrum included respondents who answered *rather likely*, *very likely*, and *definitely will* intentions; the No‐spectrum included the remaining options. Respondents were further subdivided, based on their implementation in Wave 2, into those who (1) implemented the intended measures; (2) implemented new measures that were not intended; and (3) did not implement any new measures after Wave 1 (see Table G1, Appendix G). The realized intention was calculated as the percentage of intention‐implemented respondents over the total respondents of the Yes‐spectrum. The IBG was calculated as 100% minus the realized intention.

#### 2.4.2 Assessing the Role of Intention Strength

The influence of intention strength on behavior was assessed based on the number of newly implemented measures (i.e., intended and unintended new implementation) in Wave 2 for each respondent in each intention level stated in Wave 1. The Kruskal‒Wallis test was applied (Ostertagová et al. 2014) due to the nonnormality of the newly implemented measures (Appendix D). Pairwise comparisons (Dunn test) were then applied to identify which intention strength levels were significantly different from each other, adjusting for *p* values using Bonferroni correction. The magnitude of the effect sizes was assessed based on Cohen (1992) (Appendix E).

#### 2.4.3 Comparing Predictors of Intention and Behavior

A total of 369 panelists without any no‐answer (NA) were used to compare predictors of intention and behavior. Panel datasets are inherently hierarchical; therefore, they can have two sources of variance originating from (1) the differences within a respondent at each time point compared to that respondent's average trend (random effects) and (2) different predictors assumed to have fixed unknown effects on the whole population (fixed effects) (Cernat 2023; West et al. 2022). Gelman and Hill (2007) recommend always including random effects in hierarchical models because they can capture differences that are not included in group‐level predictors.

**FIGURE 2:**
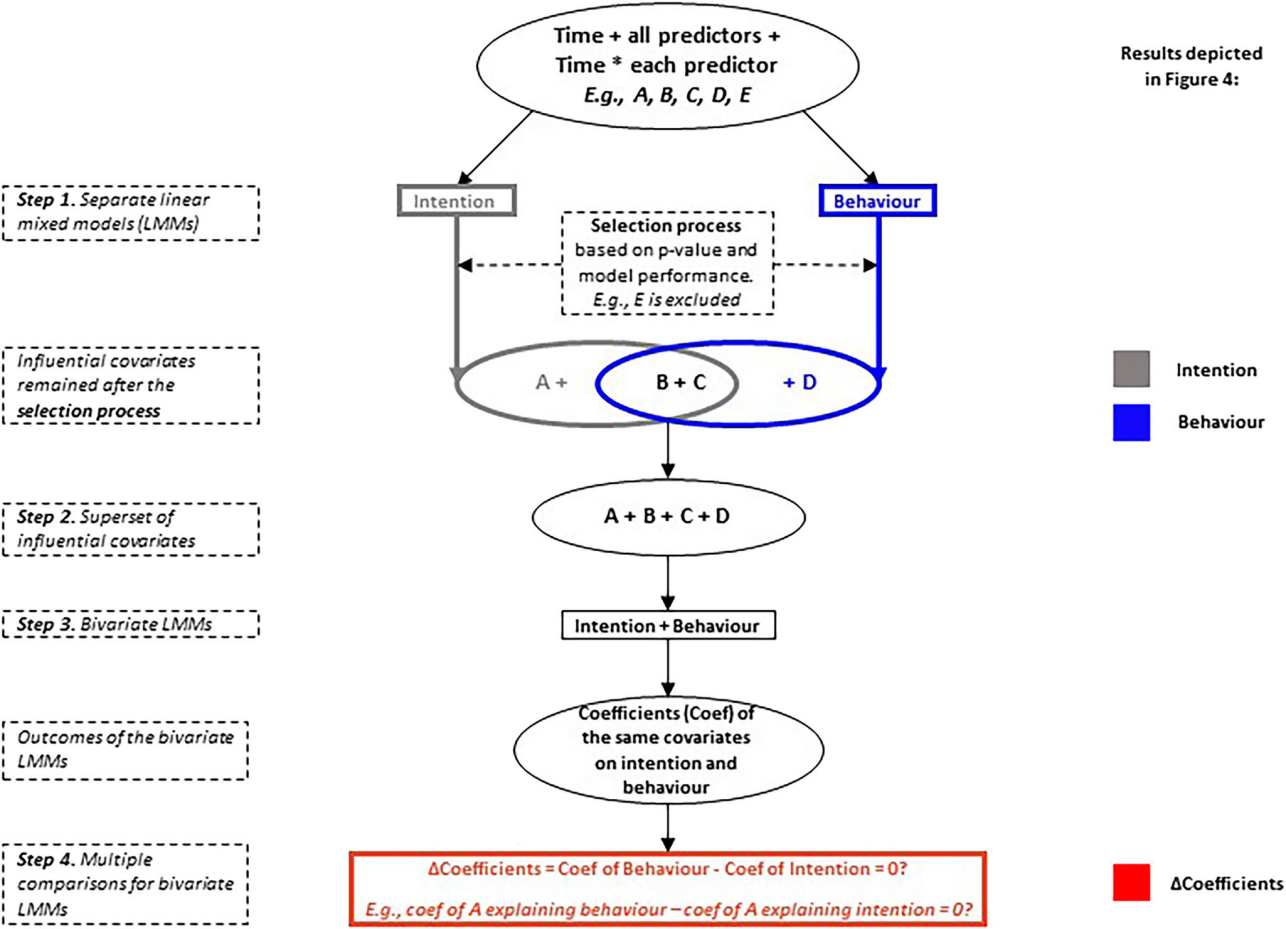
Statistical procedure to compare predictors of intention and behavior: fitting separate linear mixed models to select important covariates (Step 1), resulting in a superset of influential covariates (Step 2) that were fitted into a bivariate LMM (Step 3). The outcomes of the bivariate LMMs were then fitted into the multiple comparisons (Step 4) to compare the coefficients of the same predictors on intention and behavior. The right side indicates how the results of each step are reported in Figure 4 (Section 3). The procedure was identical and independently implemented for preparing devices and retrofitting homes.

Linear mixed models (LMMs) are a specific type of hierarchical model with group‐structured data (Gelman and Hill 2007; West et al. 2022). LMMs are better at dealing with uncertainty in data and less biased if some conditions (e.g., sphericity) are not met compared to other tests for repeated‐measure data, such as the paired *t*‐test (Nicenboim et al. 2024; Vasishth et al. 2023). LMMs are therefore applied in our research with varying‐intercept random effects (Vasishth et al. 2023) to compare predictors of intention and behavior. The statistical procedures are depicted in Figure 2.

First, influential covariates were identified by fitting all predictors and their interaction terms with time into four separate LMMs and running through a selection process to select the influential covariates for behavior and intention of each measure (Step 1). The selection process follows a top‐down approach (Cheng et al. 2010) based on *p* values (< 0.05) and model performance (West et al. 2022). *Time* (i.e., Wave 1 or 2) is controlled to be a predictor in all models (Cernat 2023).

Second, predictors for comparison were identified by taking the superset of influential covariates of intention and behavior (Step 2). These predictors were then fitted into a bivariate LMM with two outcome variables (i.e., intention and behavior) (Mehtätalo and Lappi 2020) (Step 3). Finally, coefficients of the same predictors explaining intention and behavior from the bivariate model were compared in terms of effect sizes, deploying multiple comparisons with adjusted *p* values (Bretz et al. 2010) (Step 4, Figure 2). Q‒Q plots show that although there are some violations on the extremities, the values near the medians are well described by a normal distribution. Further details and normality assumption checks are provided in Appendix F.

## 3 Results

### 3.1 The Size of the IBG

Sixty‐four percent and 89% of the 381 panelists implemented at least one specific measure for preparing devices (e.g., buying life jackets) and retrofitting homes (e.g., raising the floor), respectively. In Wave 1, 24% and 25% of the panelists stated some level of intention to implement new measures; however, 96.2% and 91.7% of the panelists had not realized their intentions for preparing devices and retrofitting homes 6 months later, respectively (Table 1).

**TABLE 1:** Respondents segmented by decisional intention and implementation (as explained in Section 2.4.1. See Table G1, Appendix G for segmentation by intention strength).

	Preparing devices				Retrofitting homes			
Stated intention	Intention realized	New implement without intent	No new implement	Total	Intention realized	New implement without intent	No new implement	Total
No‐spectrum	0	7	269	276	0	23	273	296
Yes‐spectrum	4	4	97	105	7	5	72	84
Realized (%)	3.8				8.3			
IBG (%)	96.2				91.7			

### 3.2 The Influence of Intention Strength

**FIGURE 3:**
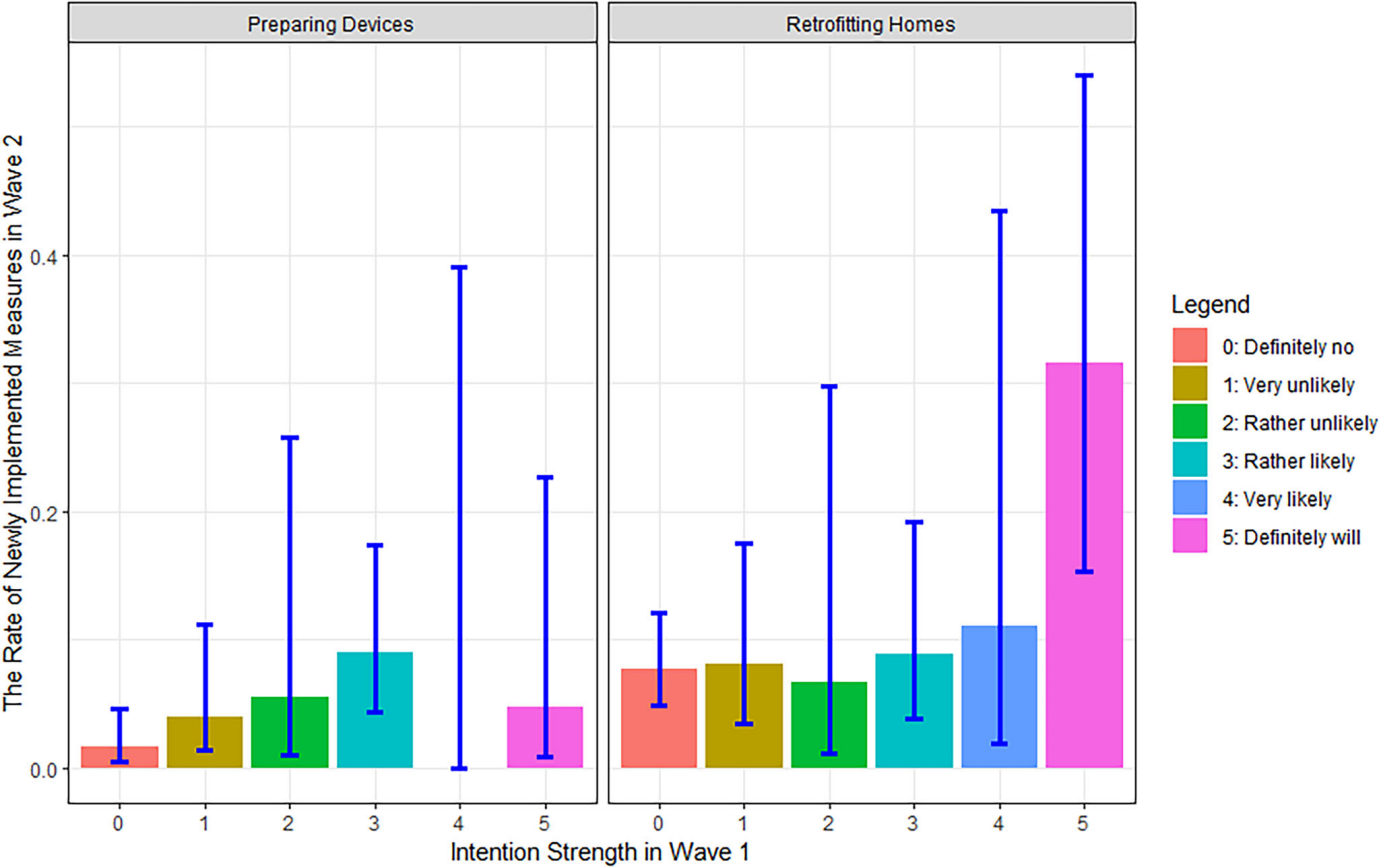
The rate of newly implemented measures for each intention strength. The error bars reflect the 95% Wilson confidence interval.

Overall, the rates of newly implemented measures for different intention levels indicate that implementation increases with intention strength (Figure 3). In particular, respondents with the strongest intention (*definitely will*) to retrofit homes have the highest rate, which is clearly distinguished from the rest. Nevertheless, the error bars show high uncertainties for the rates of *rather unlikely*, *very likely*, and *definitely will* intentions for both adaptation measures due to their low sample sizes.

The overall Kruskal‒Wallis test (Table 2) shows that new implementation of emergency devices does not differ significantly among the intention levels. This is further confirmed in the post hoc test, where all adjusted *p* values of pairwise comparisons are insignificant (p > 0.05) (see Table G2, Appendix G).

**TABLE 2:** Kruskal‒Wallis overall and pairwise test results for different intention strengths. Statistically significant results, at least at the *p* < 0.1 level, are shown in this table.

	Overall test	Pairwise test		
	Chi‐squared	Group 1	Group 2	Effect size^a^
Preparing devices	8.27			
		Definitely no (184)^b^	Rather likely (78)	0.173^‡^
Retrofitting homes	12.48^*^			
		Definitely no (219)	Definitely will (19)	0.226^**^
		Very unlikely (62)	Definitely will	0.348^*^
		Rather likely (56)	Definitely will	0.340^*^

*Note*: See Table G2, Appendix G for the full results.

^a^ See Appendix E.

^b^ Sample size.

**, *, and ^‡^ significant at *p* < 0.01, *p* < 0.05, and *p* < 0.1, respectively

In contrast, respondents with different intention levels significantly differ in implementing new activities for retrofitting homes. Specifically, the pairwise tests show significant differences between respondents with the strongest intention (*definitely will*) and those with *definitely no*, *very unlikely*, or *rather likely* intentions, with medium effect sizes (Table 2).

### 3.3 Comparing Predictors of Intention and Behavior

**FIGURE 4:**
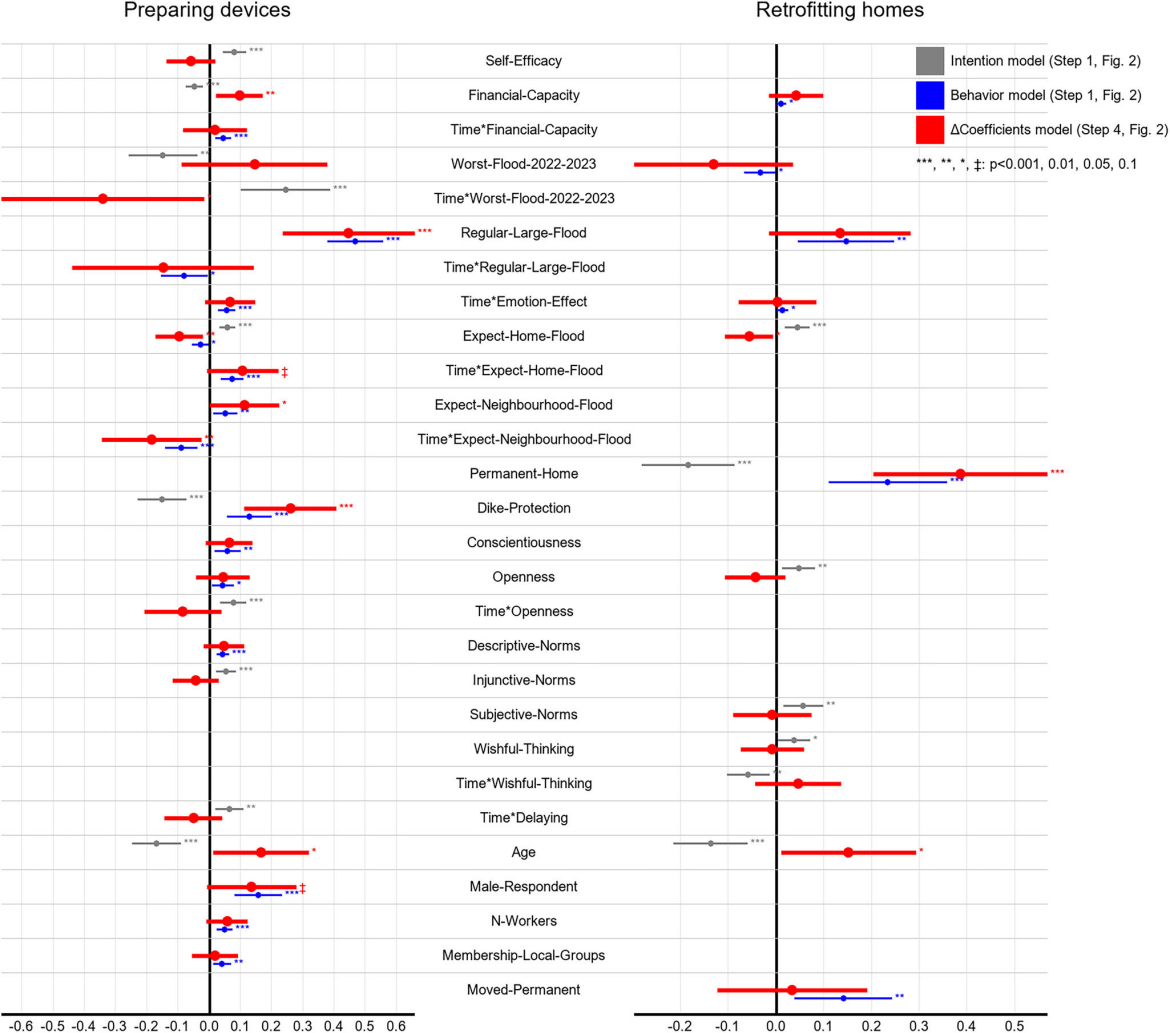
95% confidence intervals (CIs) of influential covariates and the differences in coefficients of the same predictors on intention and behavior. The covariates listed in the middle of the figure are the supersets of influential factors resulting from the selection process (Steps 1 and 2 in Figure 2) of both measures, that is, preparing devices (left) and retrofitting homes (right). Influential covariates for the models of intention and behavior (Step 1 in Figure 2), as well as the difference of coefficients (Step 4 in Figure 2), are depicted with gray, blue, and red lines, respectively.

Influential predictors and their comparisons are depicted in Figure 4, followed by guidance for interpretation and descriptions of each adaptation measure group. Details on all models are provided in Appendix H. Information on how the results link to the statistical analyses is also provided in Figure 2.

Each covariate may be influential for one measure but not necessarily for the other. For example, Self‐Efficacy^5^ was kept in the model explaining intention but was excluded from the model explaining the behavior of preparing devices after the selection process. Therefore, Self‐Efficacy is depicted with a gray line showing the 95% CI of its effect size on intention, but no blue line. Because Self‐Efficacy was kept in the intention model, it was included in the superset of influential covariates (Step 2 in Figure 2) that was fitted to the bivariate LMM of intention and behavior of preparing devices (Step 3 in Figure 2). Therefore, the difference of coefficients (ΔCoefficient) of Self‐Efficacy on intention and behavior was calculated to compare its effects on intention and behavior for preparing devices (Step 4 in Figure 2). This ΔCoefficient is depicted by the red line of Self‐Efficacy in preparing devices in Figure 4. Self‐Efficacy, however, was excluded from both intention and behavior models of retrofitting homes; therefore, it was not included in the superset for the bivariate LMMs, and there was no comparison for Self‐Efficacy in retrofitting homes.

Predictors that have only main effects (i.e., not interacted with time) remained in the models, indicating that their effects are for the whole sample across all waves. For example, Self‐Efficacy has only the main effect remaining in the intention model of preparing devices, whereas the interaction with time (i.e., Time*Self‐Efficacy) was excluded. This means that respondents with one unit higher in Self‐Efficacy will likely have 0.081 standardized units higher in intention in general (i.e., both waves).

Predictors that have the interaction terms with time remaining in the models require different interpretations: the interaction terms show the rate of change over time of the outcome variable that the predictors cause, whereas their main effects (if remaining) show the influence of the predictors at Wave 1 only (Cernat 2023). For example, Expect‐Home‐Flood has its main effect, and the interaction term with time (i.e., Time*Expect‐Home‐Flood) remained in the behavior model of preparing devices. This means that respondents who had a unit higher of Expect‐Home‐Flood had a lower implementation of 0.028 units at Wave 1; however, their implementation increased by 0.074 units over time.

A predictor's effect on intention and behavior is compared based on the effect size of its delta coefficient (ΔCoefficient): The closer to zero the point estimate of the ΔCoefficient, the more likely the predictor has similar coefficients on intention and behavior, thus unlikely causing the IBG; conversely, zero excluded from or included but very close to the edge of the confidence interval of the ΔCoefficient suggests different coefficients on intention and behavior, thus likely contributing to the IBG.

#### 3.3.1 For Preparing Devices

Among 72 input covariates of the separate LMMs (except time), only 10 and 15 remained as influential covariates for the intention and behavior model, respectively.

Among the remaining covariates, four significantly correlate with both intention and behavior: Financial‐Capacity correlates with lower intention in general but increases implementation over time (shown in Time*Financial‐Capacity). Similarly, homes perceived as being protected by dikes (Dike‐Protection) generally decrease intention but increase implementation. Conversely, the expectation of homes becoming flooded (Expect‐Home‐Flood) generally increases the intention, whereas it decreases implementation in Wave 1 but increases implementation over time. Only openness seems to increase both intention (over time, shown in Time*Openness) and implementation (in general).

Five factors significantly correlate with intention but not behavior: Self‐Efficacy and injunctive norms generally increase intention, whereas age reduces it. Perceived worst flood event in 2022 or 2023 (Worst‐Flood‐2022‐2023) and Delaying increase the intention over time.

Eight covariates significantly correlate with behavior but not with intention: higher expectation of neighborhood flooding (Expect‐Neighborhood‐Flood) and residing in communes where large floods occur regularly (Regular‐Large‐Flood) are associated with higher implementation at Wave 1 but lower implementation over time. Notably, the effect of Regular‐Large‐Flood at Wave 1 (+0.469) is much higher than that over time (−0.08). The emotional effect caused by the worst flooding event (Emotion‐Effect), Descriptive‐Norms, Conscientiousness, Male‐Respondent, the number of households' main laborers (N‐Workers), and membership of local groups or associations (Membership‐Local‐Groups) generally increase the implementation.

Overall, among 23 covariates in the superset, eight have significantly different effects on behavior compared to intention (*p* < 0.05): Financial‐Capacity, Expect‐Neighborhood‐Flood, Regular‐Large‐Flood, Expect‐Home‐Flood, Time*Expect‐Neighborhood‐Flood, Time*Worst‐Flood‐2022‐2023, Dike‐Protection, and Age. Only Time*Financial‐Capacity and Membership‐Local‐Groups have point estimates very close to the zero‐line, indicating that they likely have similar effect sizes on intention and behavior.

#### 3.3.2 For Retrofitting Homes

Among 70 input covariates of the separate LMMs (except time), only 7 and 6 remained as influential covariates for the intention and behavior model, respectively.

Among the remaining covariates, one significantly correlates with both intention and behavior, but in opposite directions: having a Permanent‐Home reduces the intention while increasing the implementation.

Five covariates significantly correlate with intention but not with behavior: Expect‐Home‐Flood, Subjective‐Norms, and Openness generally increase the intention, whereas Age reduces it; Wishful‐Thinking increases the intention at Wave 1 but reduces it over time.

Five covariates significantly correlate with behavior but not intention: Financial‐Capacity, Regular‐Large‐Flood, and moved permanently to avoid flooding (Moved‐Permanent) increase the behavior in general, while Emotion‐Effect increases it over time. In contrast, Worst‐Flood‐2022‐2023 generally reduces the behavior.

Overall, among 12 covariates in the superset, three have significantly different effects on behavior compared to intention: Permanent‐Home, Age, and Expect‐Home‐Flood. Time*Emotion‐Effect, Subjective‐Norms, and Wishful‐Thinking seem to have similar effect sizes.

## 4 Discussion

### 4.1 Large IBGs

Our research reveals large IBGs over 6 months, which are much higher than other IBGs reported in the FRD (Bubeck et al. 2020; Noll 2023; Osberghaus et al. 2025) or in the health domain (Paschal and Thomas 2016). There are several potential reasons for the comparatively large IBGs in our study.

First, the abovementioned studies surveyed over longer periods, for example, 1.5–8 years. The IBGs in our study might have been smaller if measured over a longer period, especially for more expensive measures such as raising floors. Bubeck et al. (2020) indeed found that very few people implemented new adaptation measures during the first 9 months after a severe flood in Germany, possibly because people were still recovering, whereas implementation almost doubled during 10–20 months afterward.

Second, whereas Bubeck et al. (2020), Noll (2023), and Osberghaus et al. (2025) could only elicit the intention of respondents who did not take the measures in the past, we calculated the IBG for all respondents. If respondents who have previously taken a measure have a larger IBG, our averaged sample would also reflect a larger IBG.

Third, the recent memory of the severe 2022 flooding and/or surveying contemporaneously with visible flooding (Wave 1 was during a flooding season) might produce increased flood salience. Flood salience might yield temporarily inflated intention and/or incur an obligation to overreport intention from the respondents. Hence, the intention expressed in Wave 1 may be higher than the average actual intention during the interval between Waves 1 and 2.

In contrast, the 2024 heat wave might hinder the effort to implement the intended actions. Furthermore, the heatwave might also reduce the engagement of respondents during the interviews, leading to an underreporting of the number of implemented measures. The reduced engagement in Wave 2 may be reflected in the lower recall rate of the implemented measures as well as the subjective experience of the interviewing team.

Finally, the measures taken might be fungible. While people might have the intention to take certain measures in Wave 1, when an opportunity is presented, they may opt to take another measure that is not counted as a realized intention. We, like Osberghaus et al. (2025), indeed observe new implementation without previously stated intention at nonnegligible rates (see Table G1). The unintended implementation in our study could be due to annual flooding, which shapes a flood‐knowledgeable society. In the context of a flood‐knowledgeable society, copying behavior (i.e., descriptive norms) is a rather sensible approach to flood mitigation that does not require careful planning on the part of individuals (T. T. Luu et al. 2025).

### 4.2 Intention Strength Seems to Matter

Although intention strength has no significant effect on new implementations of preparing devices, the strongest level of intention (i.e., *definitely will*) significantly increases the implementation rate of retrofitting homes compared to no intention or, more interestingly, weak intention. The significant difference between *definitely will* and *rather likely* intentions implies that if we had used dummy decisional intention, as is commonly done in the FRD (Bubeck et al. 2020; Noll 2023; Osberghaus et al. 2025), the differences within the Yes‐spectrum intention would have been obfuscated.

The general trend in Figure 3 is consistent with a monotonic increase (i.e., the dependent variable never decreases as the independent variable increases). A monotonic increase with nonzero steps would mean that all intention strengths are different from each other and that our ability to detect a difference depends on the difference's size and the sample size. For preparing devices, low rates of new implementation, especially in conjunction with small samples for *rather unlikely*, *very likely*, and *definitely will*, yield low power. It is therefore conceivable that implementation differs based on intention strength, although we have not been able to demonstrate this statistically. For retrofitting homes, the high uncertainty in the error bars of *rather unlikely* and *very likely* suggests that we might see significant differences between these intention levels and the strongest intention if the samples were larger. Based on the large differences and significant findings, a monotonic increase with a large delta coefficient between *rather likely* and *very likely* or *very likely* and *definitely will* seem most plausible.

The mixed role of intention is somewhat reflected in the aforementioned studies investigating decisional intentions in the FRD: Noll (2023) finds that intention has a positive but insignificant effect on behavior, and it is uncertain to what degree intention results in action; Osberghaus et al. (2025) find that intention contributes little to the goodness‐of‐fit of behavior models; Osberghaus et al. (2025) and Bubeck et al. (2020) find that the IBG is lower for measures that require careful planning, such as structural measures. Indeed, preparing devices in our study falls into the category of simple measures, whereas retrofitting homes is a structural measure. Stated intention is perhaps a better predictor for measures that require deliberate planning, while being less meaningful for measures that can be simply implemented.

Our findings are partly in line with studies on physical activities, suggesting that strong intentions better predict behavior (Conner and Norman 2022; Rhodes and Rebar 2017).

### 4.3 Few Shared vs. Various Incongruent Predictors

Since the influence of a predictor is best discussed in consideration of both main effect and interaction with time, we do not separate the discussion into similar and incongruent predictors. Instead, we structure the discussion following the groups of predictors. Overall, we found a few shared but various incongruent covariates of intention and behavior (Figure 4). This differs from Osberghaus et al. (2025), where the predictors of intention and behavior are broadly aligned. This could be because the studies differ in the factors investigated, statistical procedures, research areas (e.g., annual vs. rare flooding), and adaptation levels.

#### 4.3.1 Perceived Behavioral Controls

Perceived behavioral control factors are hypothesized to directly influence both intention and behavior in the TPB (Ajzen 1991) and significantly modulate the intention‒behavior relationship in empirical studies (Conner and Norman 2022). Among these factors, we find that Self‐Efficacy increases intention, while being insignificant for the behavior of preparing devices. The different influence of Self‐Efficacy on intention and behavior is insignificant, although the probability of a zero difference is rather low. This suggests that Self‐Efficacy might not have the same effects on intention and behavior; it is thus unlikely to cause the IBG. This finding does not contradict the literature in the FRD: van Valkengoed et al. (2024) find that Self‐Efficacy is directly positively correlated with intention and behavior, while Noll et al. (2020) find no evidence for the different effects of Self‐Efficacy, and Osberghaus et al. (2025) find that Self‐Efficacy hardly correlates with the size of the IBGs. Self‐Efficacy modulates the relationship between intention and behavior in physical exercise (Rhodes et al. 2022) and pro‐environmental consumption, although with small effect sizes (Grimmer and Miles 2017).

In contrast, in our study, financial capacity unexpectedly correlates with a lower intention to prepare devices at Wave 1. It, however, increases the behavior of preparing devices over time and of retrofitting homes in general, suggesting that financial capacity is a motivating factor of actual implementation but not intention. These findings contrast with behavioral theories (Ajzen 1991; Rogers 1975). One explanation is an interaction between the two measure groups: respondents with a higher financial capacity are likely to have better housing situations, thus reducing the intention to prepare emergency devices. Our dataset supports this hypothesis, as Figure I1 (Appendix I) shows that respondents with permanent houses also have higher perceived financial capacity to prepare emergency devices.

#### 4.3.2 Flood Experience

Flood experience shows mixed effects in our research. Experiencing the worst flood recently (Worst‐Flood‐2022‐2023) increases the intention of preparing devices over time, but not the implementation. It also correlates with lower implementation of retrofitting homes in general. These results suggest that recent large floods might promote intention but not actual implementation. This could be because respondents had not yet recovered from the flood within the relatively short period of 6 months to 1.5 years (Bubeck et al. 2020).

In contrast, Regular‐Large‐Flood does not correlate with the intention across adaptation measures. It, however, is strongly associated with higher implementation of preparing devices at Wave 1, whereas it reduces the implementation over time, although with a much lower effect size (i.e., 0.469 vs. 0.08). It also increases the implementation of retrofitting homes in general. These findings suggest that experiencing large floods regularly promotes actual adaptation actions. Nevertheless, the recent decrease in adaptive activities shows an ongoing change in adaptation behavior in these areas. This trend might be explained by the so‐called levee‐effects (i.e., people prepare little as they feel protected) found in higher‐income contexts (Di Baldassarre et al. 2018; Haer et al. 2020).

The findings show that Regular‐Large‐Flood and Worst‐Flood‐2022‐2023 influence intention and behavior differently; thus, they might contribute to the IBG. Data on the intention to implement adaptation measures of respondents who have just experienced severe floods within a year may not accurately predict their actual implementation. In contrast, emotional effects caused by the worst flood (Emotion‐Effect) consistently increase the actual implementation over time. The relationship between Emotion‐Effect and actual implementation should thus be tested in future studies.

The literature shows mixed findings: flood experience does not correlate strongly with behavior in van Valkengoed and Steg (2019); in contrast, it is more influential for behavior than for intention in Osberghaus et al. (2025), while it increases both intention and behavior in Richert et al. (2017). Osberghaus et al. (2025) also find that flood experience contributes to a closing of the IBG.

#### 4.3.3 Risk Perceptions

Factors related to risk perceptions show significantly different effects on intention and behavior in our data. In particular, Expect‐Home‐Flood generally increases the intention but reduces the implementation of preparing devices at Wave 1 and increases the implementation over time. This could be explained by the reverse effects of implemented measures. Due to annual flooding, some respondents might have already prepared emergency devices before our first survey. The prepared devices might give them a feeling of safety, which lowers their perceived threat from flooding, including their home being flooded, leading to the negative correlation observed at Wave 1 (Bubeck, Botzen, and Aerts 2012). However, those who have not prepared devices would still have high risk perceptions and might consequently have prepared new devices after Wave 1, leading to a positive correlation with implementation over time. Our research thus provides hints to support the well‐deployed assumption of reverse effects being present and suggests that risk perception is still an important predictor of implementation.

Mixed findings on the role of risk perceptions have been reported: Whereas risk perceptions increase intention in van Valkengoed and Steg (2019), they are less or insignificant for behavior in various studies (Lo 2013; Noll et al. 2020; Osberghaus et al. 2025; Richert et al. 2017). Bubeck, Botzen, and Aerts (2012) show that the correlation between risk perceptions and behavior is observed in 9 of 16 reviewed studies, although two of them have marginal effect sizes. Two meta‐analyses in the health domains and the FRD found that the correlations of risk perceptions with behavior are positive (Brewer et al. 2007) and even stronger than those with intention (Bamberg et al. 2017).

#### 4.3.4 Housing Situation

Similar to risk perceptions, factors related to housing situations show significantly different effects on intention and behavior. Specifically, Dike‐Protection and Permanent‐Home reduce the intention across measures but increase the implementation of emergency devices and retrofitting homes, respectively, indicating that respondents with positive housing situations have higher adaptation behavior. Permanent or dike‐protected houses lowering the intentions seem logical, whereas increasing implementations seem odd, even contradictory to the levee‐effects. This might be because people from annually flooded areas in our study are better prepared and rely on multilevel protection strategies. In the past, when economic conditions were insufficient for costly measures such as dikes, governments allocated emergency devices to these areas. Local officials provided the first author with evidence documenting the allocation of life jackets. These government measures might be a pathway to unintended adaptation. Thus, the positive correlation might be only for past implementation, which is in line with the hypothesized reverse effects of implemented measures.

Alternatively, dikes in higher‐income countries, such as the Netherlands, are heavy structures with high protection standards; that is, they also protect against low‐probability floods. In contrast, flooding occurs regularly in Central Vietnam despite some existing dike infrastructure; thus, dikes might not provide a sense of safety to reduce adaptation behavior. This reasoning also seems relevant to other lower‐income contexts, such as Bangladesh, which lack the resources to build very high dikes.

Another reason is that there might be a confounder of positive housing situations and active adaptation, such as a conscientious personality or wealth. Conscientious people tend to be well organized and effective (Rhodes et al. 2022), thus being more wealthy and better prepared to adapt to flooding. This hypothesis is supported by our findings that conscientiousness increases device preparation.

#### 4.3.5 Personality

Conscientiousness and openness generally increase the behavior of preparing devices, while openness increases the intention of preparing devices over time and of retrofitting homes in general. The influence of conscientiousness is somewhat reflected in the health domain, where conscientiousness is consistently found to modulate the relationship between intention and behavior (Conner and Norman 2022; Rhodes et al. 2022), but little is known about FRD.

#### 4.3.6 Social Norms

Descriptive norms increase the behavior of preparing devices, while injunctive and subjective norms increase the intention to prepare devices and retrofit homes, respectively. The ΔCoefficients of all social norms on intention and behavior are insignificant. In particular, the estimate of subjective norms' ΔCoefficient for retrofitting homes is nearly zero, suggesting that subjective norms have similar effects on intention and behavior.

#### 4.3.7 Maladaptation and Sociodemographic Factors

Maladaptive factors, for example, Wishful‐Thinking and Delaying (Appendix A), appear to be influential only for intention. In contrast, sociodemographic factors, except age, motivate implementation. Age is consistently found to reduce intention, but is insignificant for behavior across adaptation measures. This means that older people might express less intention to adapt, but they might not implement less than younger people. Perhaps older people have already implemented adaptation measures before. The effects of age on intention and behavior differ significantly, suggesting that age might cause IBG. In contrast, Membership‐Local‐Groups, Male‐Respondent, N‐Workers, and Moved‐Permanent are only important for behavior. The role of age on the IBG is unexpected, but other sociodemographic factors are reflected in several studies, where they are found to be unimportant in explaining the IBGs in the FRD (Osberghaus et al. 2025) and the health domain (Conner and Norman 2022).

### 4.4 Limitations

The analysis applying LMMs based on a two‐wave dataset has several limitations. First, the shape of the change was assumed to be linear. However, Bubeck et al. (2020) considered an almost 4‐year period and found that the flood adaptation behavior is nonlinear. Second, the most complex LMM, where intercepts and slopes are varied and correlated (Barr et al. 2013), could not be fitted due to a lack of observations. The rate of change in the variation between the respondents in our models was hence assumed to be constant (nonvarying slopes), and only the values at the beginning of the study differed (varying intercepts). Our models thus cannot benefit from all the advantages of the LMM. Nonetheless, using the LMM is still preferable over other repeated‐measure analyses (see Section 2.4.3).

Third, due to the large number of factors examined in the selection process to identify influential covariates, there may be issues of Type I error. This means that not all significant effects in the separate LMMs (Step 1) are true. Nevertheless, each individual significant factor in the models of preparing devices is likely to have a true correlation, while it is approximately 50% for retrofitting homes. Fourth, each intention strength has a small sample size, which, combined with the skewness of the new implementations, might limit the statistical power of Kruskal‒Wallis tests. A post hoc calculation^7^ showed that the power of the tests was within the acceptable range.

Finally, this research is different from the existing limited number of studies on IBG (Bubeck et al. 2020; Noll 2023; Osberghaus et al. 2025) in multiple ways, which should be considered when generalizing the results. For example, many respondents have repeatedly experienced lethal flooding and lived through high mortality floods or heard of them (e.g., the 1978, 1980, and 1988 floods) from elders. Although mortality rates due to flooding have dropped in recent decades, deaths still occur. The anticipation of threats to life might engender patterns of intention and behavior different from those in regions where flood deaths are exceptional, and losses are usually limited to property damage. Other differences include location, flood frequency, and questionnaires. For example, our study had a highly significant finding that the perception of being protected by a dike has a different impact on the intention to prepare devices than on the actual behavior. To the best of our knowledge, no other study has investigated this relationship. The numerous reasons listed above could all cause this finding to be absent in higher‐income countries with infrequent flooding. Until more studies on the IBG are performed, particularly in lower‐income countries, we can merely speculate on whether this finding also holds in other contexts.

### 4.5 Implications

Adaptation strategies should focus more on factors that show significantly stronger effects on behavior than intention, such as flood experience and financial capacity. However, factors that seem to be related to the reverse feedback effects caused by implemented measures, such as risk perceptions and housing situation, should be carefully reconsidered.

Our findings are also useful for flood risk assessment studies that integrate adaptation behavior in risk models using survey data. This is, for instance, done in the field of sociohydrology (Fuchs et al. 2017) by applying agent‐based models (Berghäuser 2024) that capture human decision‐making on adaptation. Due to the limited effects of stated intention and the various incongruent predictors, we recommend that data on intention be carefully reconsidered before using it as a proxy for implementation or to draw policy guidance.

Nevertheless, the use of data on intention might still be useful in certain circumstances. The opposite effects of risk perceptions and housing situation on intention and behavior in our research, which provide hints to support the reverse effects, suggest that intention may still be useful to mitigate the reverse effects of implemented measures. Given the positive and significant correlation between the highest intention strength for retrofitting homes, future studies using intention to take out the reverse effect should measure intention strength and consider the strongest intention as a proxy for behavior.

The study of the intention‒behavior relationship is still in its infancy. Measurements and definitions of intention, behavior, and the IBG vary across papers (Bubeck et al. 2020; Noll 2023; Osberghaus et al. 2025). Future studies could systematize the terminology and measurement for better comparability.

In this study, the IBG is measured by the percentage of intended actions that are unrealized. This means that intention is assumed to be constant between the two survey waves. This assumption might not be true since human intention likely changes over time. The IBG, therefore, could be measured differently, considering the intention's fluctuations, which is unfortunately currently unavailable in the FRD. Future research could thus investigate the temporal dynamics of intention to better calculate the IBG.

In addition, the percentage of new implementations that were not preceded by prior intention has not been scrutinized in our paper. While this percentage is lower than in Osberghaus et al. (2025), it is still substantial (calculation could be inferred from Table G1). Future studies could explore this as another aspect of the IBG.

Our findings also suggest that behavioral theories could benefit from reconsidering the primary role of intention and different pathways leading to intention and behavior. For example, flood experiences are found to be especially important for behavior rather than intention. Thus, they should have a direct pathway to behavior rather than through intention in models predicting adaptation behavior. Our study, nevertheless, is insufficient to determine how context‐specific the deviation from the behavioral theories that we have detected is. Future studies in other contexts could provide further knowledge to adjust the theories.

As one of the first studies to investigate the role of intention strength in FRD and to find evidence of the influence of the strongest intention on behavior in an annual flooding context, we strongly recommend that future research explore the role of intention strength in different flooding situations. Moreover, reverse feedback effects have been deployed to explain several unexpected results in our study, although they have not yet been proven. Further statistical examination of the feedback effects is, therefore, especially important.

The panel survey deployed in our research not only helped reveal the influential factors but also detected and adjusted inconsistencies, as well as unfolded potential feedback effects caused by implemented measures. However, full LMMs could not be used in our research due to a lack of observations. We thus recommend that future research deploy longitudinal surveys with more waves and suitable retention strategies and apply full LMMs to study flood adaptation behavior. Those with limited observations could apply Bayesian LMM, which can handle the full model even with only two waves (Nicenboim et al. 2024).

The seasonal changes due to the sequence of interviews (Wave 1 during a flood season and Wave 2 during a dry season) could be considered milder versions of the 2022 flood and 2024 heat wave. While considering the effect of seasonal changes would be interesting, it is unattainable for our study. To scientifically establish any seasonal effect separately from the disturbances of extreme events, politics, and other time‐bound events, a longitudinal studywith multiple waves per year over several years would be recommended.

## 5 Conclusions

This research employs a two‐wave panel survey of 401 respondents in Central Vietnam to investigate the relationship between flood adaptation intention and behavior, including the IBG, the predictive reliability of stated intention strength on behavior, and shared versus incongruent predictors of intention and behavior. Systematic correction was carefully applied to adjust inconsistencies in the dataset. In line with previous literature, we find substantial IBGs. Although intention strength seems unimportant for the behavior of preparing emergency devices, the strongest intention significantly increases the implementation of retrofitting homes. A few factors, such as emotional effects after the worst flooding, seem to have similar effects on the intention and behavior of retrofitting homes. In contrast, multiple factors, such as risk perceptions, age, perceived financial capacity, flood experience, and housing situations, are found to have different effects on the intention and behavior of preparing devices and retrofitting homes. Communication strategies aimed at increasing adaptation are thus recommended to focus on predictors that are beneficial for behavior, including financial capacity, flood experience, housing situations, descriptive norms, conscientiousness, and sociodemographic factors. Research using intention to avoid the reverse effects of implemented measures should utilize the intention strength concept. Finally, longitudinal surveys with careful retention strategies are recommended in other flooding contexts to adjust and thus increase the predictive power of behavioral theories.

## Author Contributions

**Tang T. Luu**: conceptualization, data curation, formal analysis, investigation, methodology, project administration, software, visualization, writing – original draft. **Annegret H. Thieken**: conceptualization, funding acquisition, resources, supervision, validation, writing – review and editing. **Toon Haer**: supervision, validation, writing – review and editing. **Tran Thi Tuyen**: investigation, project administration, writing – review and editing. **Philip Bubeck**: conceptualization, resources, supervision, validation, writing – review and editing.

## Funding

Tang T. Luu was supported by the German Academic Exchange Service (DAAD) [grant numbers “Research Grants—Doctoral Programmes in Germany, 2022/23 (57588370)”]. The field survey was funded by the University of Potsdam and implemented in collaboration with Vinh University, Vietnam.

## Conflicts of Interest

The authors declare no conflicts of interest.

## Supporting information




**Supplementary Information Appendix A**. List of variables and their short names (where applicable).
**Supplementary Information Appendix C**. Data processing.
**Supplementary Information Appendix D**. Normality test of the newly implemented measures.
**Supplementary Information Appendix E**. Effect sizes of the Kruskal‒Wallis test.
**Supplementary Information Appendix F**. Further details of comparing predictors.
**Supplementary Information Appendix G**. Detailed statistical results of the IBG and the Kruskal‒Wallis test.
**Supplementary Information Appendix H**. Detail statistics and interpretation of the influential factors and coefficient comparison.
**Supplementary Information Appendix I**. Boxplot showing the correlation between housing situation and the financial capacity of preparing devices.

## Endnotes

1 Informed consents were obtained for all participants to do the interviews.

2 3.24% of respondents were unable to participate because they had passed away, were ill, or had temporarily migrated, and 1.75% refused to participate in Wave 2.

3 The constructs in the PMT and TPB, although different in names, describe similar underlying concepts. We use the terms interchangeably between the TPB and PMT to be consistent with the literature.

4 These variables include newly built‐flood‐adapted houses, moving permanently, and homes perceived to be protected by dikes.

5 The names of the covariates are written with capital letters in the caption as well as the main text, where possible, to be consistent with the figure to facilitate the paper's readability.

7 The probability of correctly rejecting the null hypothesis is at least 93.5% for the obtained effect sizes of the significant Kruskal‒Wallis pairwise tests (data not shown).
