# Supplementary material for: Exploring the Intention–Behavior Relationship in Flood Adaptation Using Longitudinal Data
Source: Risk Anal. 2026 May 11;46:e70261. doi: 10.1111/risa.70261 (PMC13158897; doi:10.1111/risa.70261)
Supplement: Supplementary file 1 — Supplementary Information Appendix A. List of variables and their short names (where applicable). Supplementary Information Appendix C. Data processing. Supplementary Information Appendix D. Normality test of the newly implemented measures. Supplementary Information Appendix E. Effect sizes of the Kruskal‒Wallis test. Supplementary Information Appendix F. Further details of comparing predictors. Supplementary Information Appendix G. Detailed statistical results of the IBG and the Kruskal‒Wallis test. Supplementary Information Appendix H. Detail statistics and interpretation of the influential factors and coefficient comparison. Supplementary Information Appendix I. Boxplot showing the correlation between housing situation and the financial capacity of preparing devices. [file RISA-46-0-s001.docx]

**Supplementary files**

**Exploring the Intention–Behaviour Relationship in Flood Adaptation Using Longitudinal Data**

**APPENDICES**

**Appendix A. List of Variables and Their Short Names (where applicable)**

Table A1. List of variables used in the current research (the order of variables follows the same order in the survey questionnaire)

| **Short name** | **Variables/ References** | **Question and answering scales** |  |
| --- | --- | --- | --- |
|  |  |  |  |
|  | ***Dependent variables*** | |  |
|  | **Retrofitting homes** | **Have you already retrofitted your house to adapt to flooding?** |  |
|  | Bubeck et al. 2013; Poussin et al. 2014; Binh et al. 2020; Kreibich et al. 2015 | No |  |
|  |  | Yes |  |
|  | **Specific measures of retrofitting homes (if the answer for the previous question is “Yes”).** | **What did you do to retrofit your house and when did you do it ? (Multiple answers are possible):** |  |
|  |  | Elevating the entrance |  |
|  |  | Elevating the floor |  |
|  |  | Replacing the floor, or wall, or roof by (better) water-proof materials |  |
|  |  | Preparing a garret inside the existing house |  |
|  |  | Elevating the kitchen |  |
|  |  | Expanding the yard or garden |  |
|  |  | Building a flood-proof hut aside from the main house |  |
|  |  | Others (measures that were not mentioned in the list above) |  |
|  | **Intention strength to retrofitting homes** | **How likely is it that you intend to (further) retrofit your house to adapt to flood in the next 5 months?** |  |
|  |  | Definitely no |  |
|  |  | Very unlikely |  |
|  |  | Rather unlikely |  |
|  |  | Rather likely |  |
|  |  | Very likely |  |
|  |  | Definitely will |  |
|  |  | **What do you intend to do to (further) retrofit your house?** |  |
|  |  | Elevating the entrance |  |
|  |  | Elevating the floor |  |
|  |  | Replacing the floor, or wall, or roof by (better) water-proof materials |  |
|  |  | Preparing a garret inside the existing house |  |
|  |  | Elevating the kitchen |  |
|  |  | Expanding the yard or garden |  |
|  |  | Building a flood-proof hut aside from the main house |  |
|  |  | Others (measures that were not mentioned in the list above) |  |
|  | **Preparing devices** | **Have you already prepared flood emergency devices?** |  |
|  | Poussin et al. 2014; Binh et al. 2020 | No |  |
|  |  | Yes |  |
|  | **Specific measures of preparing devices (if the answer for the previous question is “Yes”).** | **Which flood-emergent devices did you prepare? (Multiple answers are possible):** |  |
|  |  | Handmade rafts using trees such as banana or bamboos |  |
|  |  | Handmade rafts using barrels |  |
|  |  | Barrels |  |
|  |  | Wooden rafts |  |
|  |  | A boat |  |
|  |  | Life jackets |  |
|  |  | Shelfs |  |
|  |  | Others (measures that were not mentioned in the list above) |  |
|  | **Intention strength to preparing devices** | **How likely is it that you intend to (further) prepare more flood-emergent devices in the next 5 months?** |  |
|  |  | Definitely no |  |
|  |  | Very unlikely |  |
|  |  | Rather unlikely |  |
|  |  | Rather likely |  |
|  |  | Very likely |  |
|  |  | Definitely will |  |
|  |  | **Which items do you intend to (further) prepare?** |  |
|  |  | Handmade rafts using trees such as banana or bamboos |  |
|  |  | Handmade rafts using barrels |  |
|  |  | Barrels |  |
|  |  | Wooden rafts |  |
|  |  | A boat |  |
|  |  | Life jackets |  |
|  |  | Shelfs |  |
|  |  | Others (measures that were not mentioned in the list above) |  |
|  | ***Independent variables*** | |  |
| **Descriptive-Norms** | **Descriptive norms for retrofitting homes** | **Most of the people you know retrofit their house to adapt to flood** |  |
|  | Bubeck et al. 2013; Lo 2013; Poussin et al. 2014; van Valkengoed and Steg 2019; Le et al. 2014; Tuu et al. 2008; Chung and Rimal 2016 | Disagree completely |  |
|  |  | Disagree |  |
|  |  | Disagree slightly |  |
|  |  | Agree slightly |  |
|  |  | Agree |  |
|  |  | Agree completely |  |
| **Descriptive-Norms** | **Descriptive norms for preparing emergency devices** | **Most of the people you know prepare flood-emergent devices** |  |
|  | Bubeck et al. 2013; Lo 2013; Poussin et al. 2014; van Valkengoed and Steg 2019; Le et al. 2014; Tuu et al. 2008; Chung and Rimal 2016 | Disagree completely |  |
|  |  | Disagree |  |
|  |  | Disagree slightly |  |
|  |  | Agree slightly |  |
|  |  | Agree |  |
|  |  | Agree completely |  |
| **Injunctive-Norms** | **Injunctive norms for retrofitting homes** | **Most of the people you know are of the opinion that everyone should retrofit their house to adapt to flood** |  |
|  | van Valkengoed and Steg 2019; Chung and Rimal 2016 | Disagree completely |  |
|  |  | Disagree |  |
|  |  | Disagree slightly |  |
|  |  | Agree slightly |  |
|  |  | Agree |  |
|  |  | Agree completely |  |
| **Injunctive-Norms** | **Injunctive norms for preparing emergency devices** | **Most of the people you know are of the opinion that everyone should prepare flood-emergent devices** |  |
|  | van Valkengoed and Steg 2019; Chung and Rimal 2016 | Disagree completely |  |
|  |  | Disagree |  |
|  |  | Disagree slightly |  |
|  |  | Agree slightly |  |
|  |  | Agree |  |
|  |  | Agree completely |  |
| **Subjective-Norms** | **Subjective norms for retrofitting homes** | **Most of the important people expect you to retrofit your house** |  |
|  | Lo 2013; Chung and Rimal 2016 | Disagree completely |  |
|  |  | Disagree |  |
|  |  | Disagree slightly |  |
|  |  | Agree slightly |  |
|  |  | Agree |  |
|  |  | Agree completely |  |
| **Subjective-Norms** | **Subjective norms for preparing emergency devices** | **Most of the important people expect you to prepare flood emergency devices** |  |
|  | Lo 2013; Chung and Rimal 2016 | Disagree completely |  |
|  |  | Disagree |  |
|  |  | Disagree slightly |  |
|  |  | Agree slightly |  |
|  |  | Agree |  |
|  |  | Agree completely |  |
| **Self-Efficacy** | **Self-efficacy for retrofitting homes** | **Your family has sufficient knowledge to retrofit your house to adapt to flood** |  |
|  | Grothmann and Reusswig 2006; Bubeck et al. 2013; Poussin et al. 2014; van Valkengoed and Steg 2019; Vo et al. 2021; Le et al. 2014; Bamberg et al. 2017; Binh et al. 2020 | Disagree completely |  |
|  |  | Disagree |  |
|  |  | Disagree slightly |  |
|  |  | Agree slightly |  |
|  |  | Agree |  |
|  |  | Agree completely |  |
| **Self-Efficacy** | **Self-efficacy for preparing devices** | **Your family has sufficient knowledge to prepare flood emergency devices to mitigate flood impacts** |  |
|  | Grothmann and Reusswig 2006; Bubeck et al. 2013; Poussin et al. 2014; van Valkengoed and Steg 2019; Vo et al. 2021; Le et al. 2014; Bamberg et al. 2017; Binh et al. 2020 | Disagree completely |  |
|  |  | Disagree |  |
|  |  | Disagree slightly |  |
|  |  | Agree slightly |  |
|  |  | Agree |  |
|  |  | Agree completely |  |
| **Response-Efficacy** | **Response efficacy retrofitting homes** | **Retrofitting your house helps to significantly reduce flood damage** |  |
|  | Grothmann and Reusswig 2006; Bubeck et al. 2013; Poussin et al. 2014; van Valkengoed and Steg 2019; Vo et al. 2021; Le et al. 2014; Bamberg et al. 2017; Binh et al. 2020 | Disagree completely |  |
|  |  | Disagree |  |
|  |  | Disagree slightly |  |
|  |  | Agree slightly |  |
|  |  | Agree |  |
|  |  | Agree completely |  |
| **Response-Efficacy** | **Response efficacy for preparing devices** | **Preparing the flood-emergent devices helps to significantly reduce flood damage** |  |
|  | Grothmann and Reusswig 2006; Bubeck et al. 2013; Poussin et al. 2014; van Valkengoed and Steg 2019; Vo et al. 2021; Le et al. 2014; Bamberg et al. 2017; Binh et al. 2020 | Disagree completely |  |
|  |  | Disagree |  |
|  |  | Disagree slightly |  |
|  |  | Agree slightly |  |
|  |  | Agree |  |
|  |  | Agree completely |  |
| **Financial-Capacity** | **Financial capacity for retrofitting homes** | **Your family has sufficient finance to retrofit your house to adapt to flood** |  |
|  | Grothmann and Reusswig 2006; Bubeck et al. 2013; Poussin et al. 2014; van Valkengoed and Steg 2019; Vo et al. 2021; Le et al. 2014; Bamberg et al. 2017; Binh et al. 2020 | Disagree completely |  |
|  |  | Disagree |  |
|  |  | Disagree slightly |  |
|  |  | Agree slightly |  |
|  |  | Agree |  |
|  |  | Agree completely |  |
| **Financial-Capacity** | **Financial capacity for preparing devices** | **Your family has sufficient finance to prepare flood-emergent devices** |  |
|  | Grothmann and Reusswig 2006; Bubeck et al. 2013; Poussin et al. 2014; van Valkengoed and Steg 2019; Vo et al. 2021; Le et al. 2014; Bamberg et al. 2017; Binh et al. 2020 | Disagree completely |  |
|  |  | Disagree |  |
|  |  | Disagree slightly |  |
|  |  | Agree slightly |  |
|  |  | Agree |  |
|  |  | Agree completely |  |
| **Fatalism** | **Fatalism** | **There is nothing you can do to reduce damage from flooding** |  |
|  | Grothmann and Reusswig 2006; Bubeck et al. 2013; Binh et al. 2020 | Disagree completely |  |
|  |  | Disagree |  |
|  |  | Disagree slightly |  |
|  |  | Agree slightly |  |
|  |  | Agree |  |
|  |  | Agree completely |  |
| **Wishful-Thinking** | **Wishful thinking** | **Your ancestors and/or Gods will protect you from flooding** |  |
|  | Grothmann and Reusswig 2006; Bubeck et al. 2013; Binh et al. 2020 | Disagree completely |  |
|  |  | Disagree |  |
|  |  | Disagree slightly |  |
|  |  | Agree slightly |  |
|  |  | Agree |  |
|  |  | Agree completely |  |
| **Denial** | **Denial** | **Flooding is not a real problem in your area** |  |
|  | Grothmann and Reusswig 2006; Bubeck et al. 2013; Binh et al. 2020 | Disagree completely |  |
|  |  | Disagree |  |
|  |  | Disagree slightly |  |
|  |  | Agree slightly |  |
|  |  | Agree |  |
|  |  | Agree completely |  |
| **Delaying** | **Delaying** | **You do not have the time to care about flood protection** |  |
|  | Grothmann and Reusswig 2006; Bubeck et al. 2013; Binh et al. 2020 | Disagree completely |  |
|  |  | Disagree |  |
|  |  | Disagree slightly |  |
|  |  | Agree slightly |  |
|  |  | Agree |  |
|  |  | Agree completely |  |
|  | **House ownership** | **Do you own your house?** |  |
|  | Grothmann and Reusswig 2006; Poussin et al. 2014 | No |  |
|  |  | Yes |  |
| **Permanent-Home** | **House type** | **What is the type of your house?** |  |
|  |  | Permanent multi-story house |  |
|  |  | Permanent single-story house |  |
|  |  | Semi-permanent |  |
|  |  | Temporary |  |
|  | **Building a new flood-adaptive house** | **Have you already built a new house in a flood-adaptive way, e.g., using water-proof materials, flood-proof hut, having a flood-shelter in the house, using floating materials to build floating house, etc.?** |  |
|  |  | No |  |
|  |  | Yes |  |
|  | **Frequency of flooding in general** | **How would you describe the frequency of flooding in your area in general?** |  |
|  | Bubeck et al. 2013; Grothmann and Reusswig 2006; Poussin et al. 2014; Bamberg et al. 2017 | Never flooded |  |
|  |  | Very rarely |  |
|  |  | Rarely |  |
|  |  | Frequently |  |
|  |  | Very frequently |  |
|  |  | Flooded every flood season |  |
|  | **Frequency of house get flooded** | **How often did your house get flooded in the last 10 years?** |  |
|  | Bubeck et al. 2013; Grothmann and Reusswig 2006; Poussin et al. 2014; Bamberg et al. 2017 | Not flooded at all |  |
|  |  | 1 to 2 times |  |
|  |  | 3 to 4 times |  |
|  |  | 5 to 6 times |  |
|  |  | 7 to 8 times |  |
|  |  | 9 to 10 times |  |
|  |  | More than 10 times |  |
| **Worst-Flood-2022-2023** | **Worst flood event** | **In which year did you experience the worst and most recent flooding event?** |  |
| **Emotion-Effect** | **Emotional effect after the worst flooding event** | **Overall, how bad did it feel back then when you experienced the worst flooding event?** |  |
|  | Binh et al. 2020; Bamberg et al. 2017; van Valkengoed and Steg 2019 | Not bad at all |  |
|  |  | Slightly bad |  |
|  |  | Quite bad |  |
|  |  | Bad |  |
|  |  | Very bad |  |
|  |  | Extremely bad |  |
| **Expect-Home-Flood** | **Perceived probability of home being flooded** | **How likely is it that your house will get flooded in the next 5 years?** |  |
|  | Poussin et al. 2014; Bubeck et al. 2013; Reynaud et al. 2013; van Valkengoed and Steg 2019 | Very unlikely |  |
|  |  | Unlikely |  |
|  |  | Slightly unlikely |  |
|  |  | Slightly likely |  |
|  |  | Likely |  |
|  |  | Very likely |  |
| **Expect-Neighbourhood-Flood** | **Perceived probability of neighbourhood being flooded** | **How likely is it that your neighbourhood will get flooded in the next 5 years?** |  |
|  | Poussin et al. 2014; Bubeck et al. 2013; Reynaud et al. 2013; van Valkengoed and Steg 2019 | Very unlikely |  |
|  |  | Unlikely |  |
|  |  | Slightly unlikely |  |
|  |  | Slightly likely |  |
|  |  | Likely |  |
|  |  | Very likely |  |
|  | **Expected severity** | **How severe do you think would be the level of damage to human health and safety in your family due to flooding in the next 5 years?** |  |
|  | Bubeck et al. 2013; Poussin et al. 2014; van Valkengoed and Steg 2019 | No damage at all |  |
|  |  | Very little damage |  |
|  |  | Little damage |  |
|  |  | Moderate damage |  |
|  |  | Severe damage |  |
|  |  | Very severe damage |  |
|  |  |  |  |
| **Conscientiousness** | **Conscientiousness** | **An average score of: "You see yourself as someone who does a thorough job" and the reverse of "You see yourself as someone who tends to be lazy"** |  |
|  | Rhodes et al. 2022 | Disagree completely |  |
|  |  | Disagree |  |
|  |  | Disagree slightly |  |
|  |  | Agree slightly |  |
|  |  | Agree |  |
|  |  | Agree completely |  |
| **Openness** | **Openness** | **An average score of: "You see yourself as someone who has an active imagination" and the reverse of "You see yourself as someone who has few artistic interests"** |  |
|  | Rhodes et al. 2022 | Disagree completely |  |
|  |  | Disagree |  |
|  |  | Disagree slightly |  |
|  |  | Agree slightly |  |
|  |  | Agree |  |
|  |  | Agree completely |  |
|  | **Agreableness** | **An average score of: "You see yourself as someone who is generally trusting" and the reverse of "You see yourself as someone who tends to find fault with others"** |  |
|  |  | Disagree completely |  |
|  |  | Disagree |  |
|  |  | Disagree slightly |  |
|  |  | Agree slightly |  |
|  |  | Agree |  |
|  |  | Agree completely |  |
|  | **Extraversion** | **An average score of: "You see yourself as someone who is outgoing, sociable" and the reverse of "You see yourself as someone who is reserved"** |  |
|  |  | Disagree completely |  |
|  |  | Disagree |  |
|  |  | Disagree slightly |  |
|  |  | Agree slightly |  |
|  |  | Agree |  |
|  |  | Agree completely |  |
|  | **Neuroticism** | **An average score of: "You see yourself as someone who gets nervous easily" and the reverse of "You see yourself as someone who is relaxed, handles stress well"** |  |
|  |  | Disagree completely |  |
|  |  | Disagree |  |
|  |  | Disagree slightly |  |
|  |  | Agree slightly |  |
|  |  | Agree |  |
|  |  | Agree completely |  |
| **Moved-Permanent** | **Moving permanently** | **In the past, have you already moved to another place to avoid flooding?** |  |
|  |  | No |  |
|  |  | Yes |  |
|  | **Urban commune** | **Commune where the respondent lives. The communes were then used to label whether the respondent is living in a rural or urban area.** |  |
|  | Bubeck et al. 2013 | Rural |  |
|  |  | Urban |  |
| **Dike-Protection** | **House perceived to be protected by a dike** | **Is your house protected by a river dike, river dam, or sea dike?** |  |
|  | Bubeck et al. 2013 | No |  |
|  |  | Yes |  |
| **Male-Respondent** | **Respondent’s gender** |  |  |
|  | Vo et al. 2021 | Female |  |
|  |  | Male |  |
| **Age** | **Age** | **What is your birthyear in your identification card?** |  |
|  | Poussin et al. 2014; Bubeck et al. 2013 | The birthyear was then used to calculate age of the respondent at the interviewing time |  |
|  | **Highest education level** | **What is the highest education level in your family?** |  |
|  | Ngo et al. 2020 | Primary school |  |
|  |  | Secondary school |  |
|  |  | High school |  |
|  |  | University or college |  |
|  |  | Master |  |
|  |  | Doctor and higher |  |
|  | **Gender of the household's head** | **What is the gender of your household's head?** |  |
|  | Vo et al. 2021 | Female |  |
|  |  | Male |  |
| **Membership-Local-Groups** | **Membership of local groups** | **How many local groups or associations are you a member of?** |  |
|  | Ngo et al. 2020; Vo et al. 2021 | Not a member of any group |  |
|  |  | 1 group |  |
|  |  | 2 groups |  |
|  |  | 3 groups |  |
|  |  | 4 groups |  |
|  |  | 5 groups or more than 5 groups |  |
| **N-Workers** | **Number of main labours in the household** | **How many people in your family are main labors?** |  |
| **Income** | **Total income of the household** | **Could you estimate what is the average income of your family for a month (in million VND)?** |  |
|  | Bubeck et al. 2013; Grothmann and Reusswig 2006 | The income was then transformed into the US dollar (USD) |  |

***The Creation of the Variable Regular-Large-Flood***

An additional predictor, named Regular-Large-Flood, was created to capture different flooding patterns in different communes based on the experience of the worst flooding event. Based on respondents' perceived worst flood events, the flooding pattern of the surveyed communes is divided into two types: regular (i.e., worst flood events have spread throughout the last 50 years) and recent (i.e., worst flood events mostly happened during the 2020s). Respondents from communes with regular large floods (i.e., there were multiple worst floods reported in the communes in the last 50 years) will receive a value of “*yes*”; otherwise, a value of “*no*” (Appendix B). Theoretically, this procedure could result in false negatives in case districts are regularly affected by large floods, while it has recently been hit by the heaviest flood in living memory. However, in the specific case of Nghe An, this does not appear to be the case, as evidenced by its flood history.


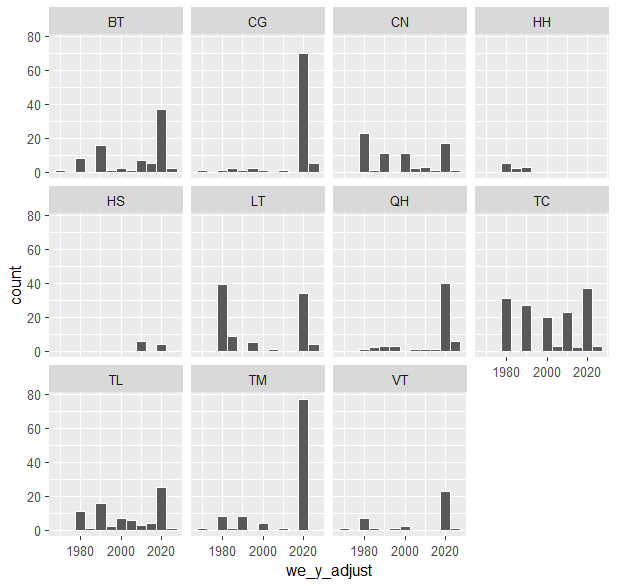


Figure B1. Years perceived as the worst flood event in the surveyed communes

Accordingly, communes and wards that experience regular large flooding events include Chau Nhan (CN), Hung Hoa (HH), Trung-Phuc Cuong (TC), and Thanh Lam (TL). Communes and wards where respondents experienced big flooding events recently are Ben Thuy (BT), Cau Giat (CG), Hong Son (HS), Long Thanh (LT), Quynh Hong (QH), Thanh My (TM), and Vinh Thanh (VT). Respondents from the regular big flood areas will receive a value of “yes” for the Regular-Large-Flood variable and a value of “no” otherwise.

**Appendix C. Data processing**

*Inconsistencies in the data*

In total, 43, 82, 81, 36, and 62 respondents have inconsistent answers for preparing devices, retrofitting homes, newly built flood-adapted houses, Moved-Permanent, and Dike-Protection, respectively. The inconsistent respondents are widely distributed among the sample (Figure C1).


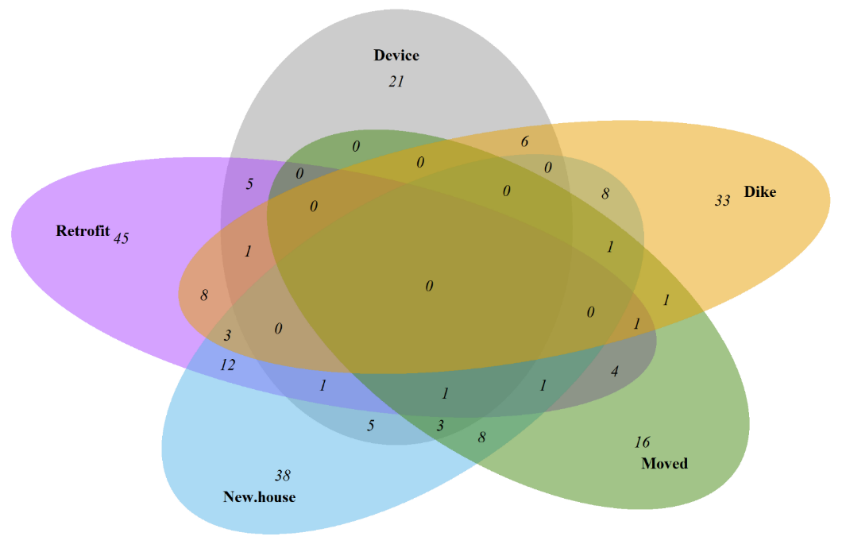


Figure C1. The number of respondents answered yes in wave 1 and no in wave 2 for the same variable. Each variable's number of inconsistencies is the sum of all numbers in its ellipse. For example, the number of respondents who prepared devices is 21 + 5 + 1 + 1 + 5 + 3 + 1 + 6 + 8*0 = 43. The overlapping parts are the number of the same respondents who have inconsistencies in different variables, e.g., 0 (in the centre).

The lack of clustering in inconsistent individuals suggests it is unlikely that: (1) certain respondents cause the inconsistency, e.g., they did not take it seriously, or they suffer from memory issues; (2) one of the enumerators caused the inconsistency; and (3) the same respondents were not tracked. The size difference (factor 2) between preparing devices and retrofitting homes suggests that (4) transcription errors are less likely to cause inconsistencies. Possible causes of the inconsistencies include: (5) the questions were poorly understood, leading to inconsistent answers; (6) the respondents forgot part of the measures that they had taken in either wave 1 or 2 (i.e., incomplete recall); and (7) hurrying interviews due to harsh conditions such as the heat wave during the wave 2 survey might exacerbate problems (5) and (6). Based on these assumptions, the inconsistencies are corrected, as explained below.

*Adjustment of the inconsistencies*

Several rules for adjustments were followed. If a specific measure was mentioned in wave 1 but not in wave 2, this measure will be added to wave 2. If a particular measure was mentioned in wave 2, but not in wave 1, and the year it was implemented was before 2023, this measure was added back to wave 1. An example of the adjustment with hypothetical answers is illustrated in Table C1.

Table C1. Example of the adjustment (red colour) for preparing devices for one hypothetical respondent.

| **Specific measure/ year implemented** | | | | | **Behaviour** |
| --- | --- | --- | --- | --- | --- |
|  | **Life jacket** | **Boat** | **Shelf** | **Raft** |  |
| ***Before adjustment*** | | | | | |
| **Wave 1** | 2019 | No | 2021 | No | 2 |
| **Wave 2** | No | 2020 | No | 2023/2024 | 2 |
| ***After adjustment*** | | | | | |
| **Wave 1** | 2019 | 2020 | 2021 | No | 3 |
| **Wave 2** | No | 2020 | 2021 | 2023/2024 | 3 |

The adjustment was implemented for specific measures related to preparing devices and retrofitting homes, except for preparing barrels, life jackets, wooden rafts, and other emergency devices, as these devices might become damaged and discarded over time. Similarly, Moved-Permanent was adjusted for both waves. However, houses perceived to be protected by a dike and newly-built-flood-adapted houses were only adjusted for wave 2 based on wave 1 because the implemented years were not obtained.

Although some respondents recalled the years differently between the two waves, especially those who experienced the worst flooding before 2000, the perceived year of the worst flood event was not adjusted because most of the deviation still fell within the same categories, i.e., regular and recent. However, missing information on the Worst-Flood-2022-2023 of 12 respondents was adjusted based on the other wave.

The average values of the behaviour indices for both groups of measures decreased over the two waves before the adjustment, but increased after the adjustment for wave 2 and changed in the opposite direction after adjusting both waves (Figure C2). Specifically, the behaviour slightly decreases for preparing devices, whereas it slightly increases for retrofitting homes. Virtually, respondents seem to vary greatly at the beginning of the study, whereas their rates of change seem to be less varied.


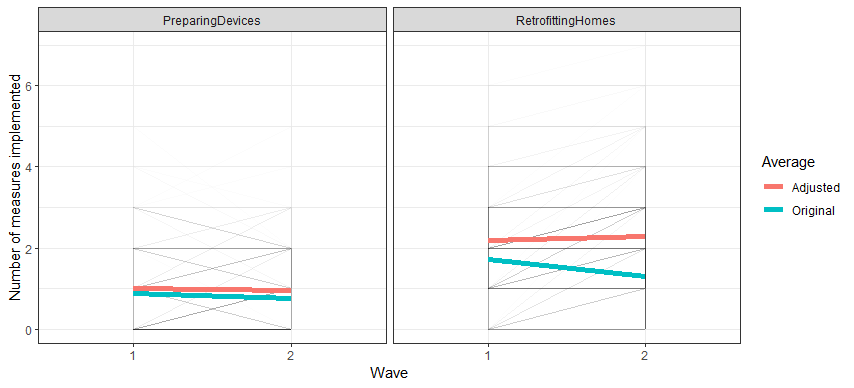


Figure C2. Changes in the average number of measures implemented before and after the inconsistency adjustment.

*Counting and inconsistencies adjustments for the “others” option*

In addition to the main specific measures of each adaptation measure group, we also asked whether the respondents had implemented any specific adaptation measures that were not listed in our questionnaires. This is because, before conducting the main survey, we implemented a pilot to collect common adaptation measures in the research areas and adjusted our questionnaires accordingly based on the measures collected. Thus, the different specific measures in the “others” in the main survey were minor, and the total number of each specific measure in the “others” was too small to make a single category. Nevertheless, the “others” measure was included in the indices of implemented measures for both preparing devices and retrofitting homes. The “others” measure in the retrofitting home was assumed to be maintained over time, thus following the same procedure of inconsistencies adjustment as other specific measures of retrofitting homes. By contrast, the “others” measure in the preparing devices was assumed not to be necessarily maintained over time; thus, an adjustment for inconsistencies was not applied.

**Appendix D. Normality Test of the Newly Implemented Measures**

Table D1. Results of the Shapiro test for normality

|  | **Test Statistic** | **p-value** |
| --- | --- | --- |
| Preparing devices | 0.19 | 2.05E-37 |
| Retrofitting homes | 0.29 | 8.43E-36 |

The p-values of preparing devices and retrofitting homes are well below 0.05, indicating that the distributions are not normally distributed.

**Appendix E. Effect Sizes of the Kruskal-Wallis Test**

The Kruskal-Wallis test was applied, with the effect sizes of each pair-wise comparison calculated following the formula:

$$r=|\frac{z}{\sqrt{N}}|$$

*Z: standardised test statistic; N: the sum of sample sizes of the two groups of the pair-wise comparisons.*

The magnitude of the effect is small when the effect is from 0.1 to less than 0.3, medium when from 0.3 to less than 0.5, and large when equal to or greater than 0.5 (Cohen, 1992).

**Appendix F. Further Details of Comparing Predictors**

*Regression selection/Linear mixed models*

Several statistical tests can be used to investigate repeated-measures data, such as the paired t-test, repeated measures ANOVA, and linear mixed model (LMM). LMM has been increasingly applied in different fields such as linguistics and biology (Vasishth et al., 2023). LMMs have several advantages over other statistical tests. First, LMMs can deal better with uncertainty in data, such as outliers or extreme data points, due to the shrinkage effect, i.e., the estimate is shrunk towards the mean value of the behaviour of the whole population, thus reducing the impact of outliers and could yield more robust estimates in case of missing values (Vasishth et al., 2023). Second, traditional methods for repeated-measures data analysis, e.g., ANOVA, could yield biased results if conditions are not met, e.g., sphericity, whereas LMMs can be less biased (Nicenboim et al., 2024). Therefore, the LMM is applied in our research to investigate the influential, shared, and incongruent predictors of intention and behaviour.

*Model-building strategy to find influential factors*

To compare coefficients of the same predictors, intention and behaviour were standardised beforehand. In addition, age was also standardised. Time is coded as 0 and 1 for the first and second waves, respectively, to facilitate interpretation: the intercept represents the value at the beginning of the study, i.e., wave 1. Respondents with NA values of the selected variables were excluded from the dataset used for the models and tests. That left 369 respondents for the LMMs.

*Coefficient comparison*

Multiple comparisons of coefficients for bivariate LMMs were applied using the glht function from the multcomp package in R, following guidance from Bretz et al. (2010) and several webpages^[[1]](#footnote-1)^). The glht function with the default approach results in smaller p-values compared to the Bonferroni test because it takes into account the correlations between the covariates (Bretz et al., 2010, p. 5).

*Checking normality assumption in univariate and bivariate linear mixed models*

Checking the normality assumption of the linear model is considered necessary in various sources (Vasishth et al., 2023; West et al., 2022) while unnecessary in others (Norman, 2010). Least Squares and Empirical Bayes approaches are commonly used to calculate the residuals (<https://cran.r-project.org/web/packages/HLMdiag/vignettes/hlm_resid.html>). Due to a deficiency in rank, we are not able to calculate Least Squares residuals. Whereas the Empirical Bayes residual is considered a limited value for checking normality, violations of normality for random effects seem to “not play a critical role in overall inferences based on linear mixed models” (West et al., 2022, p. 43). Nevertheless, we provide Normal Q-Q Plots, calculated based on the Empirical Bayes approach, for checking normality of conditional (level 1), random effects (higher level), and marginal residuals of behaviour, intention, and bivariate models of preparing devices and retrofitting homes (Figure F1). The calculation of the residuals follows guidance from and was implemented using the R package HLMdiag (<https://cran.r-project.org/web/packages/HLMdiag/vignettes/hlm_resid.html)>.


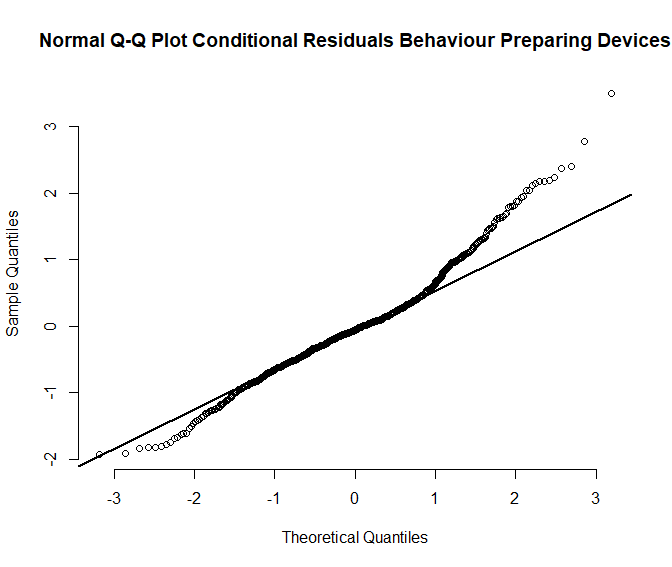

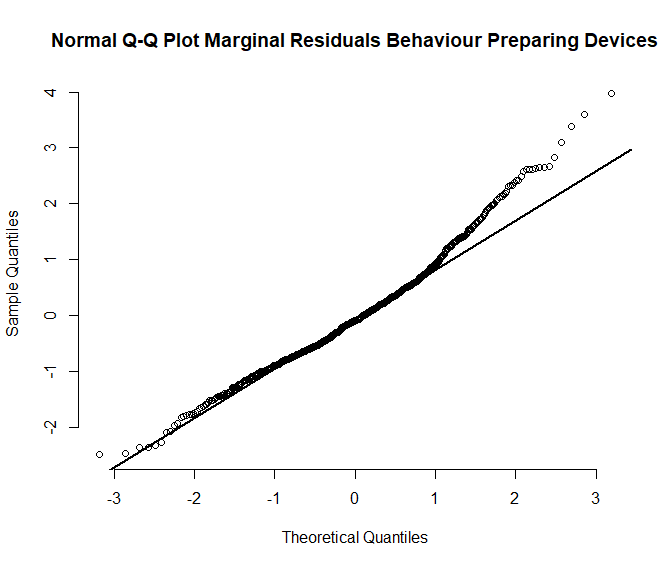

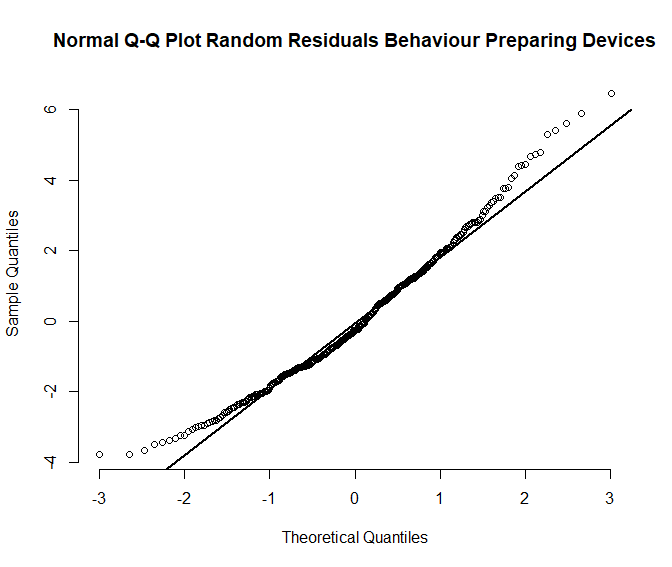

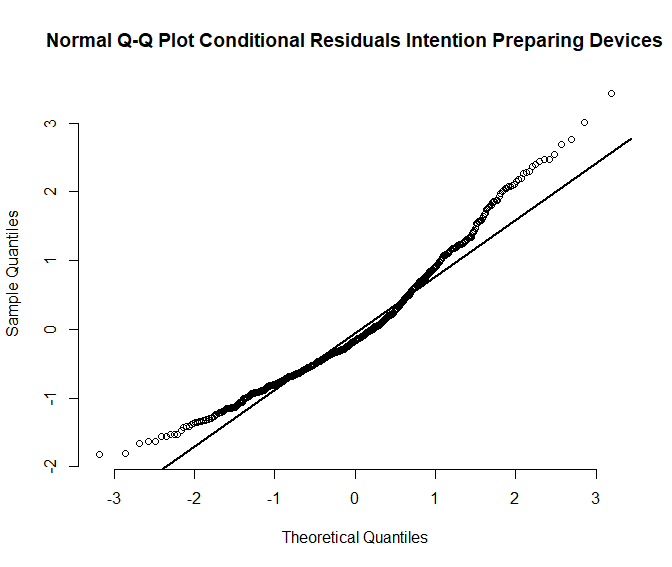

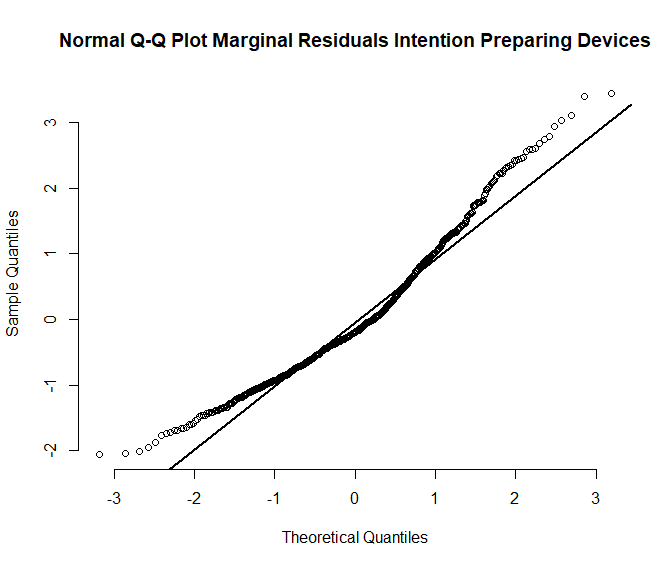

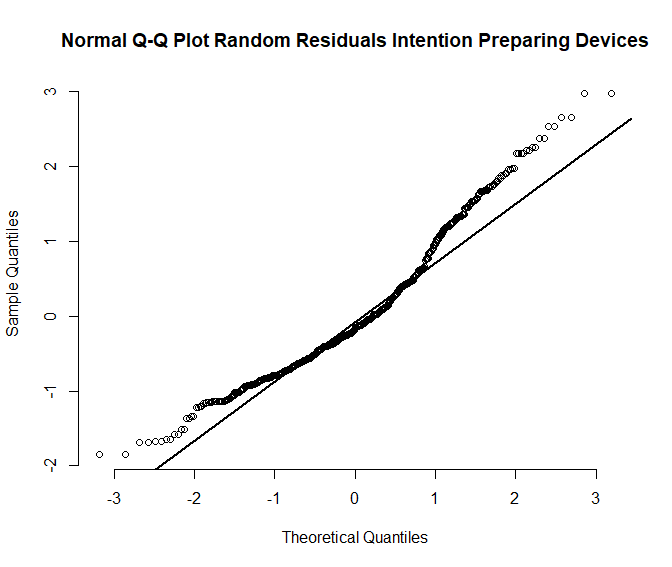

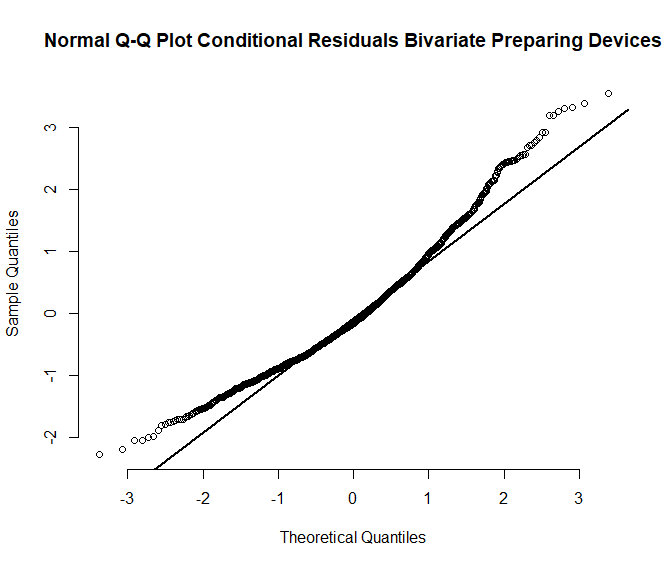

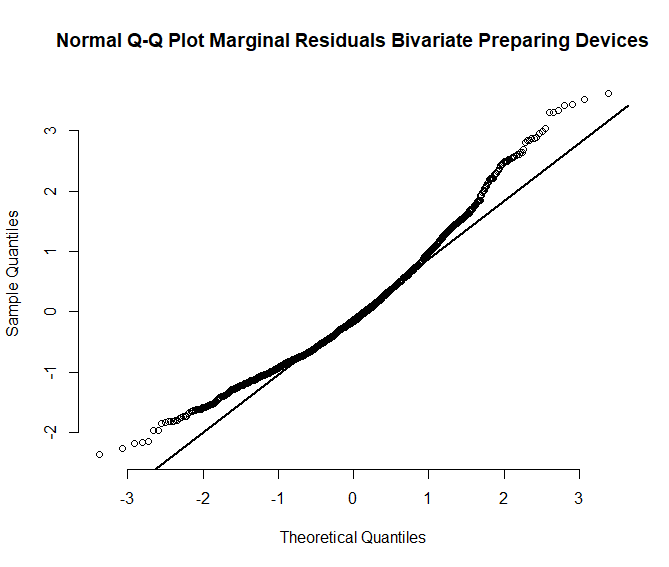

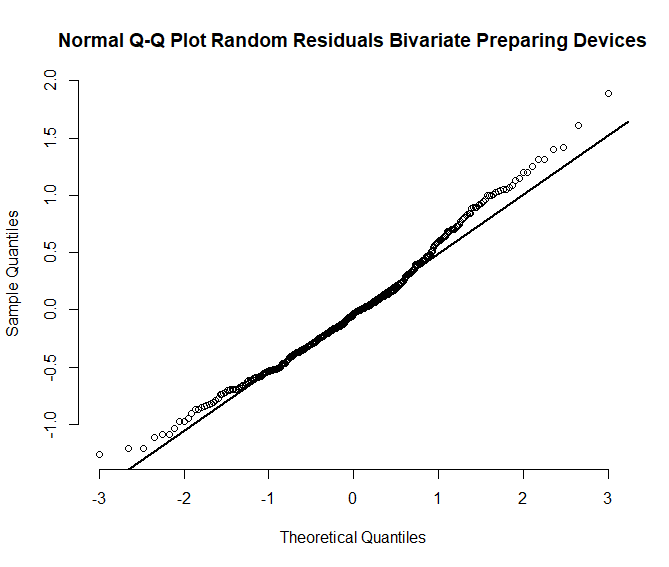

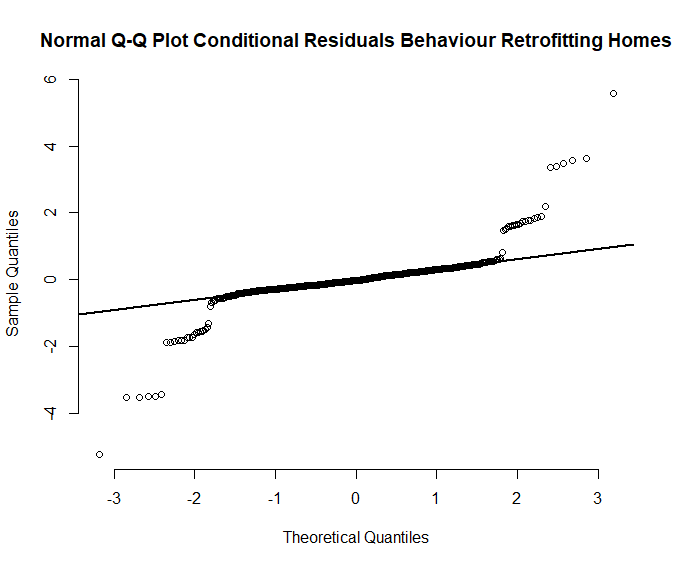

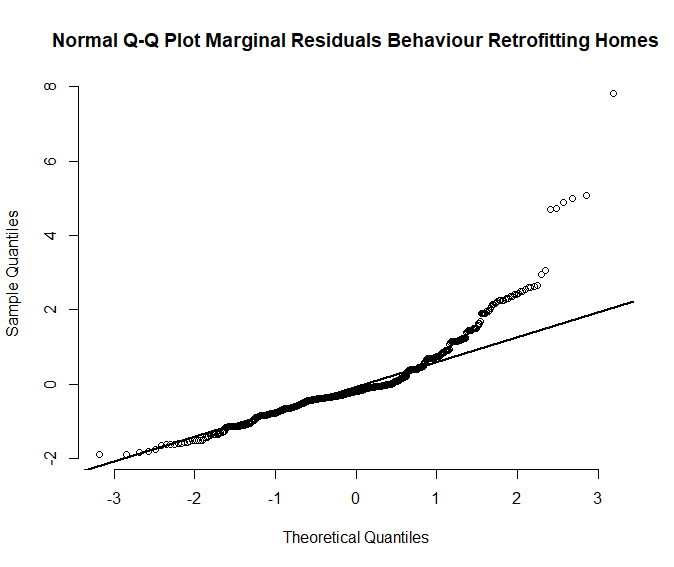

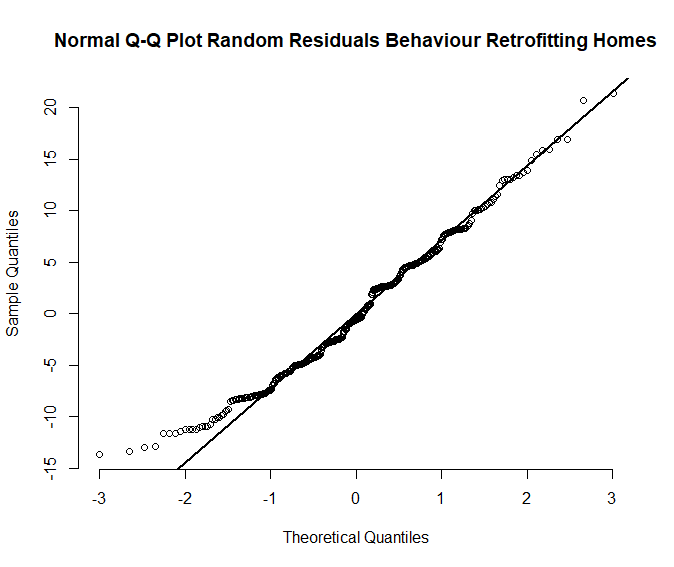


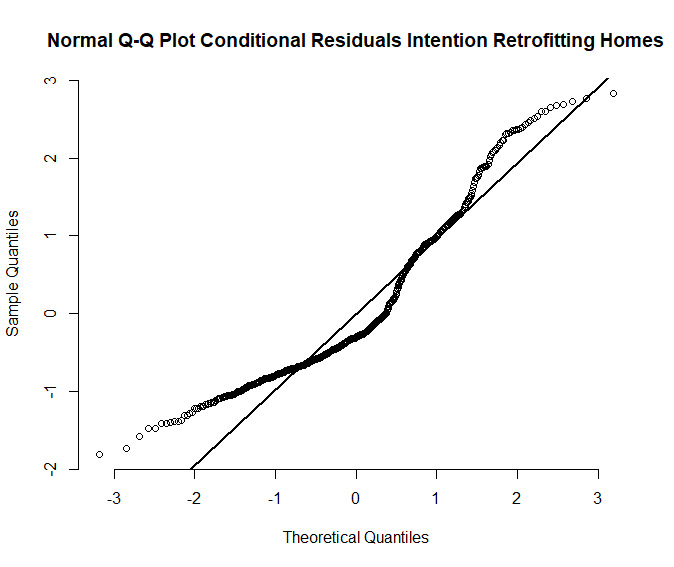

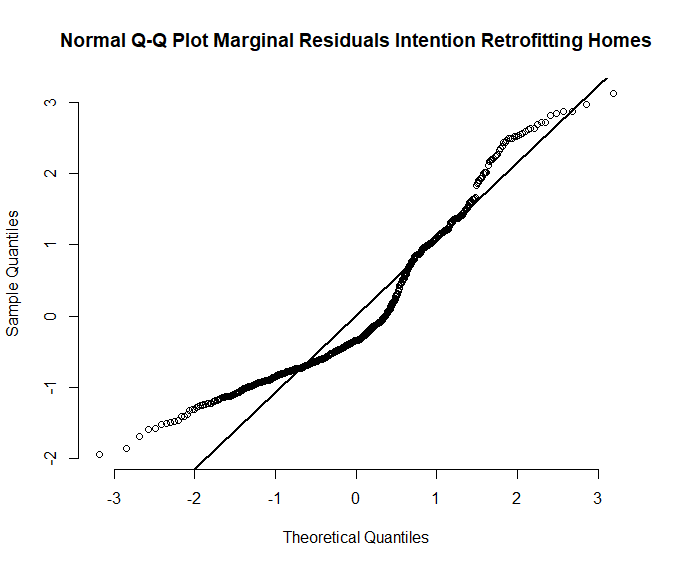

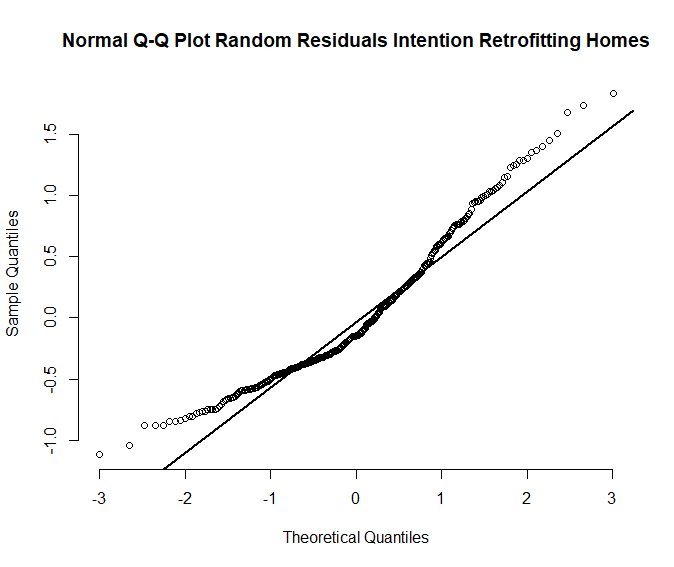

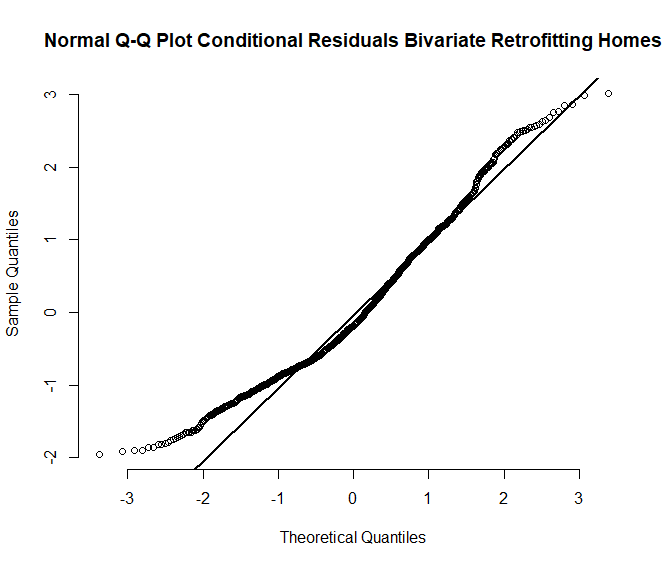

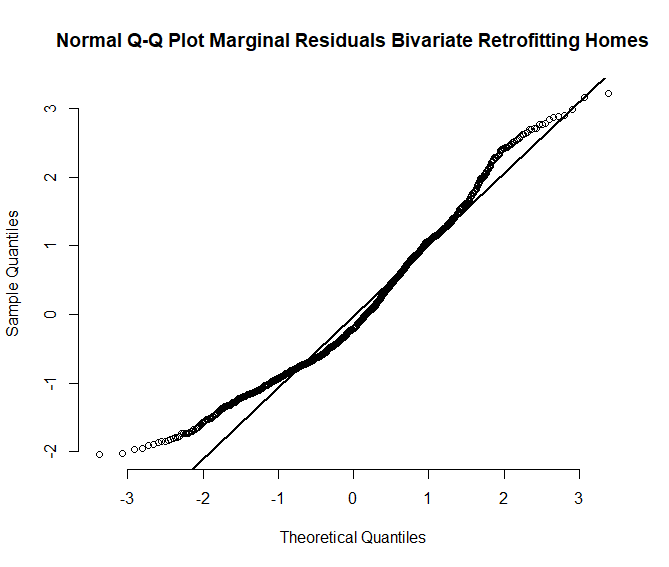

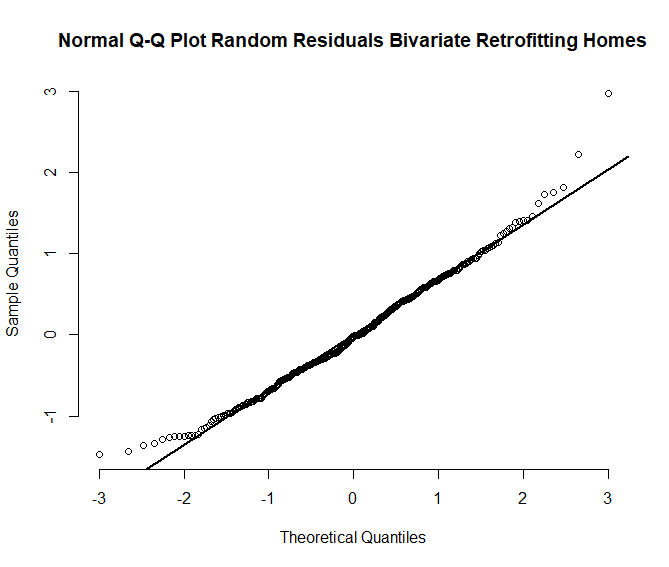


Figure F1. Normal Q-Q Plot to check normality assumptions of the linear mixed models.

**Appendix G. Detailed Statistical Results of the IBG and the Kruskal-Wallis Test**

Respondents were first classified into two spectra following six levels of intention strength in wave 1: the Yes-spectrum included respondents who answered *rather likely, very likely,* and *definitely will* intentions; the No-spectrum included rather *unlikely, very unlikely*, and *definitely no*.

Table G1. The number of respondents in different intention strengths and implementation groups.

|  | **Preparing devices** | | | | **Retrofitting homes** | | | |
| --- | --- | --- | --- | --- | --- | --- | --- | --- |
| ***Intention strength*** | ***Intention realised*** | ***New implement without intent*** | ***No new  implement*** | ***Total*** | ***Intention realised*** | ***New implement without intent*** | ***No new  implement*** | ***Total*** |
| Definitely no | 0 | 3 | 181 | *184* | 0 | 17 | 202 | *219* |
| Very unlikely | 0 | 3 | 71 | *74* | 0 | 5 | 57 | *62* |
| Rather unlikely | 0 | 1 | 17 | *18* | 0 | 1 | 14 | *15* |
| ***No-spectrum*** | ***0*** | ***7*** | ***269*** | ***276*** | ***0*** | ***23*** | ***273*** | ***296*** |
| Rather likely | 3 | 4 | 71 | *78* | 2 | 3 | 51 | *56* |
| Very likely | 0 | 0 | 6 | *6* | 0 | 1 | 8 | *9* |
| Definitely will | 1 | 0 | 20 | *21* | 5 | 1 | 13 | *19* |
| ***Yes-spectrum*** | ***4*** | ***4*** | ***97*** | ***105*** | ***7*** | ***5*** | ***72*** | ***84*** |
| NA | 0 | 0 | 0 | *0* | 0 | 0 | 1 | *1* |
| *Total* | *4* | *11* | *366* | *381* | *7* | *28* | *346* | *381* |
| **Realised (%)** | **3.8** |  |  |  | **8.3** |  |  |  |
| **IBG (%)** | **96.2** |  |  |  | **91.7** |  |  |  |

Table G2. Kruskal-Wallis with post-hoc test using Bonferroni correction. Group 1 and Group 2 columns are different levels of intention coded as 0 (definitely not) to 5 (definitely will).

| **Preparing devices** | | | | | | | | |
| --- | --- | --- | --- | --- | --- | --- | --- | --- |
| ***Group 1*** | ***Group 2*** | ***n1*** | ***n2*** | ***Statistic*** | ***p*** | ***p.adjusted*** | ***Significant level*** | ***r*** |
| 0 | 1 | 184 | 74 | 0.902 | 0.367 | 1 | ns | 0.0561 |
| 0 | 2 | 184 | 18 | 0.814 | 0.416 | 1 | ns | 0.0573 |
| 0 | 3 | 184 | 78 | 2.8 | 0.0056 | 0.0759 | ‡ | 0.173 |
| 0 | 4 | 184 | 6 | -0.201 | 0.84 | 1 | ns | -0.0146 |
| 0 | 5 | 184 | 21 | 0.696 | 0.486 | 1 | ns | 0.0486 |
| 1 | 2 | 74 | 18 | 0.293 | 0.77 | 1 | ns | 0.0305 |
| 1 | 3 | 74 | 78 | 1.57 | 0.117 | 1 | ns | 0.127 |
| 1 | 4 | 74 | 6 | -0.489 | 0.625 | 1 | ns | -0.0547 |
| 1 | 5 | 74 | 21 | 0.147 | 0.883 | 1 | ns | 0.015 |
| 2 | 3 | 18 | 78 | 0.68 | 0.497 | 1 | ns | 0.0694 |
| 2 | 4 | 18 | 6 | -0.604 | 0.546 | 1 | ns | -0.123 |
| 2 | 5 | 18 | 21 | -0.127 | 0.899 | 1 | ns | -0.0203 |
| 3 | 4 | 78 | 6 | -1.09 | 0.275 | 1 | ns | -0.119 |
| 3 | 5 | 78 | 21 | -0.888 | 0.374 | 1 | ns | -0.0893 |
| 4 | 5 | 6 | 21 | 0.527 | 0.598 | 1 | ns | 0.101 |
| **Retrofitting homes** | | | | | | | | |
| 0 | 1 | 219 | 62 | 0.0874 | 0.93 | 1 | ns | 0.00521 |
| 0 | 2 | 219 | 15 | -0.165 | 0.869 | 1 | ns | -0.0108 |
| 0 | 3 | 219 | 56 | 0.346 | 0.73 | 1 | ns | 0.0209 |
| 0 | 4 | 219 | 9 | 0.304 | 0.761 | 1 | ns | 0.0202 |
| 0 | 5 | 219 | 19 | 3.49 | 0.000487 | 0.0073 | ** | 0.226 |
| 1 | 2 | 62 | 15 | -0.197 | 0.844 | 1 | ns | -0.0225 |
| 1 | 3 | 62 | 56 | 0.213 | 0.832 | 1 | ns | 0.0196 |
| 1 | 4 | 62 | 9 | 0.255 | 0.799 | 1 | ns | 0.0303 |
| 1 | 5 | 62 | 19 | 3.13 | 0.00173 | 0.0259 | * | 0.348 |
| 2 | 3 | 15 | 56 | 0.33 | 0.742 | 1 | ns | 0.0391 |
| 2 | 4 | 15 | 9 | 0.35 | 0.726 | 1 | ns | 0.0715 |
| 2 | 5 | 15 | 19 | 2.54 | 0.011 | 0.165 | ns | 0.436 |
| 3 | 4 | 56 | 9 | 0.144 | 0.885 | 1 | ns | 0.0179 |
| 3 | 5 | 56 | 19 | 2.95 | 0.00321 | 0.0481 | * | 0.34 |
| 4 | 5 | 9 | 19 | 1.81 | 0.071 | 1 | ns | 0.341 |
| *ns, **, *, and ‡: p-value is insignificant, significant at level p<0.01, p<0.05, and p<0.1, respectively.* | | | | | | | | |

**Appendix H. Detail Statistics and Interpretation of the Influential Factors and Coefficient Comparison**

Since time is included in all LMMs (Table H1), the intercepts show the grand means of outcome variables at wave 1, controlled for other predictors in each model. The coefficient of time shows the rate of change of the outcome variables at each time step. For example, the behaviour of preparing devices is reduced by 0.215 units (p<0.05) after each passage of time (i.e., a half year in this study).

Table H1. Detailed summary of the results of the separate LMMs

| ***Code*** | ***Predictors*** | **Preparing devices** | | **Retrofitting homes** | |
| --- | --- | --- | --- | --- | --- |
|  |  | ***Behaviour*** | ***Intention*** | ***Behaviour*** | ***Intention*** |
|  | *Intercept* | *-1.099**** | *-0.426**** | *-0.331**** | *-0.544**** |
| 00.1. | Time | -0.215* | -0.375*** | -0.007 | 0.287*** |
| 01.1. | Self-Efficacy | Ex | 0.081*** | Ex | Ex |
| 01.2. | Time*Self-Efficacy | Ex | Ex | Ex | Ex |
| 02.1. | Financial-Capacity | Ex | -0.047*** | 0.01* | Ex |
| 02.2. | Time*Financial-Capacity | 0.045*** | Ex | Ex | Ex |
| 03.1. | Worst-Flood-2022-2023 | Ex | -0.148** | -0.033* | Ex |
| 03.2. | Time*Worst-Flood-2022-2023 | Ex | 0.245*** | Ex | Ex |
| 04.1. | Regular-Large-Flood | 0.469*** | Ex | 0.146** | Ex |
| 04.2. | Time*Regular-Large-Flood | -0.08* | Ex | Ex | Ex |
| 05.1. | Emotion-Effect | Ex | Ex | Ex | Ex |
| 05.2. | Time*Emotion-Effect | 0.057*** | Ex | 0.014* | Ex |
| 06.1. | Expect-Home-Flood | -0.028* | 0.058*** | Ex | 0.044*** |
| 06.2. | Time*Expect-Home-Flood | 0.074*** | Ex | Ex | Ex |
| 07.1. | Expect-Neighbourhood-Flood | 0.052** | Ex | Ex | Ex |
| 07.2. | Time*Expect-Neighbourhood-Flood | -0.09*** | Ex | Ex | Ex |
| 08.1. | Permanent-Home | Ex | Ex | 0.234*** | -0.184*** |
| 08.1. | Response efficacy | Ex | Ex | Ex | Ex |
| 08.2. | Time*Permanent-Home | Ex | Ex | Ex | Ex |
| 08.2. | Time*Response efficacy | Ex | Ex | Ex | Ex |
| 09.1. | Dike-Protection | 0.129*** | -0.151*** | Ex | Ex |
| 09.2. | Time*Dike-Protection | Ex | Ex | Ex | Ex |
| 10.1. | Conscientiousness | 0.058** | Ex | Ex | Ex |
| 10.2. | Time*Conscientiousness | Ex | Ex | Ex | Ex |
| 11.1. | Agreeableness | Ex | Ex | Ex | Ex |
| 11.1. | Openness | 0.043* | Ex | Ex | 0.047** |
| 11.2. | Time*Agreeableness | Ex | Ex | Ex | Ex |
| 11.2. | Time*Openness | Ex | 0.077*** | Ex | Ex |
| 12.1. | Descriptive-Norms | 0.044*** | Ex | Ex | Ex |
| 12.1. | Extraversion | Ex | Ex | Ex | Ex |
| 12.2. | Time*Descriptive-Norms | Ex | Ex | Ex | Ex |
| 12.2. | Time*Extraversion | Ex | Ex | Ex | Ex |
| 13.1. | Injunctive-Norms | Ex | 0.054*** | Ex | Ex |
| 13.1. | Neuroticism | Ex | Ex | Ex | Ex |
| 13.2. | Time*Injunctive-Norms | Ex | Ex | Ex | Ex |
| 13.2. | Time*Neuroticism | Ex | Ex | Ex | Ex |
| 14.1. | Subjective-Norms | Ex | Ex | Ex | 0.057** |
| 14.2. | Time*Subjective-Norms | Ex | Ex | Ex | Ex |
| 15.1. | Wishful-Thinking | Ex | Ex | Ex | 0.037* |
| 15.2. | Time*Wishful-Thinking | Ex | Ex | Ex | -0.058** |
| 16.1. | Delaying | Ex | Ex | Ex | Ex |
| 16.1. | Expected severity to human health | Ex | Ex | Ex | Ex |
| 16.2. | Time*Delaying | Ex | 0.065** | Ex | Ex |
| 16.2. | Time*Expected severity to human health | Ex | Ex | Ex | Ex |
| 17.1. | Age | Ex | -0.169*** | Ex | -0.137*** |
| 17.1. | Avoidance | Ex | Ex | Ex | Ex |
| 17.2. | Time*Age | Ex | Ex | Ex | Ex |
| 17.2. | Time*Avoidance | Ex | Ex | Ex | Ex |
| 18.1. | Male-Respondent | 0.157*** | Ex | Ex | Ex |
| 18.2. | Time*Male-Respondent | Ex | Ex | Ex | Ex |
| 19.1. | N-Workers | 0.049*** | Ex | Ex | Ex |
| 19.1. | Not a real problem (denial) | Ex | Ex | Ex | Ex |
| 19.2. | Time*N-Workers | Ex | Ex | Ex | Ex |
| 19.2. | Time*Not a real problem (denial) | Ex | Ex | Ex | Ex |
| 20.1. | Membership-Local-Groups | 0.042** | Ex | Ex | Ex |
| 20.2. | Time*Membership-Local-Groups | Ex | Ex | Ex | Ex |
| 21.1. | Moved-Permanent | Ex | Ex | 0.141** | Ex |
| 21.2. | Time*Moved-Permanent | Ex | Ex | Ex | Ex |
| 22.1. | Newly built flood-adapted house | Ex | Ex | NI | NI |
| 22.2. | Time*Newly built flood-adapted house | Ex | Ex | NI | NI |
| 24.1. | Urban commune | Ex | Ex | Ex | Ex |
| 24.2. | Time*Urban commune | Ex | Ex | Ex | Ex |
| 26.1. | Home ownership | Ex | Ex | Ex | Ex |
| 26.2. | Time*Home ownership | Ex | Ex | Ex | Ex |
| 28.1. | Frequency of flooding in general | Ex | Ex | Ex | Ex |
| 28.2. | Time*Frequency of flooding in general | Ex | Ex | Ex | Ex |
| 29.1. | Frequency of house get flooded | Ex | Ex | Ex | Ex |
| 29.2. | Time*Frequency of house get flooded | Ex | Ex | Ex | Ex |
| 30.1. | Male household's head | Ex | Ex | Ex | Ex |
| 30.2. | Time*Male household's head | Ex | Ex | Ex | Ex |
| 32.1. | Highest education level | Ex | Ex | Ex | Ex |
| 32.2. | Time*Highest education level | Ex | Ex | Ex | Ex |
| 36.1. | Participation in local groups | Ex | Ex | Ex | Ex |
| 36.2. | Time*Participation in local groups | Ex | Ex | Ex | Ex |
| *Criterion (%)* | | | | | |
|  | ICC | 66.25 | 27.71 | 96.14 | 14.53 |
|  | Marginal R2 | 33.78 | 17.98 | 7.97 | 9.58 |
|  | Conditional R2 | 77.65 | 40.71 | 96.45 | 22.72 |
| *Ex: Excluded after the model selection: NI: Not included in the input of the models in the first place.* | | | | | |
| ****, **, and *: p-value is significant at level p<0.001, p<0.01, and p<0.05, respectively.* | | | | | |

Table H2. Detailed statistics of the final models of the separate LMMs, after the selection process (Step 1 in Figure 2).

| **Intention model of preparing devices** |  |  |  |  |  |
| --- | --- | --- | --- | --- | --- |
| Predictor | Estimate | Std. Error | df | t value | Pr(>\|t\|) |
| (Intercept) | -0.42570464 | 0.09965424 | 677.5502 | -4.271817 | 2.216317e-05 |
| 00.1. Time | -0.37477386 | 0.06863972 | 585.8949 | -5.460014 | 7.041079e-08 |
| 01.1. Self-Efficacy | 0.08108457 | 0.01862310 | 671.9966 | 4.353978 | 1.545322e-05 |
| 02.1. Financial-Capacity | -0.04708942 | 0.01365420 | 683.8597 | -3.448713 | 5.978145e-04 |
| 03.1. Worst-Flood-2022-2023 | -0.14789897 | 0.05531064 | 658.2780 | -2.673969 | 7.681931e-03 |
| 03.2. Time*Worst-Flood-2022-2023 | 0.24501430 | 0.07223183 | 499.1452 | 3.392054 | 7.487921e-04 |
| 06.1. Expect-Home-Flood | 0.05832295 | 0.01238360 | 680.4733 | 4.709691 | 3.008146e-06 |
| 09.1. Dike-Protection | -0.15114486 | 0.03915064 | 482.3573 | -3.860598 | 1.285163e-04 |
| 11.2. Time*Openness | 0.07725414 | 0.02101869 | 638.9903 | 3.675497 | 2.573201e-04 |
| 13.1. Injunctive-Norms | 0.05443384 | 0.01547565 | 677.5145 | 3.517385 | 4.649183e-04 |
| 16.2. Time*Delaying | 0.06527197 | 0.02209851 | 642.5234 | 2.953683 | 3.254840e-03 |
| 17.1. Age | -0.16853141 | 0.03925133 | 390.9414 | -4.293649 | 2.219507e-05 |
| **Behviour model of preparing devices** |  |  |  |  |  |
| Predictor | Estimate | Std. Error | df | t value | Pr(>\|t\|) |
| (Intercept) | -1.09899437 | 0.123591594 | 594.3607 | -8.892145 | 7.155042e-18 |
| 00.1. Time | -0.21514532 | 0.104425511 | 439.2927 | -2.060276 | 3.996059e-02 |
| 02.2. Time*Financial-Capacity | 0.04499716 | 0.012269137 | 452.7261 | 3.667508 | 2.740264e-04 |
| 04.1. Regular-Large-Flood | 0.46892848 | 0.044945105 | 528.9541 | 10.433360 | 2.662488e-23 |
| 04.2. Time*Regular-Large-Flood | -0.07967023 | 0.037505027 | 334.8932 | -2.124255 | 3.438118e-02 |
| 05.2. Time*Emotion-Effect | 0.05651123 | 0.013330862 | 469.8812 | 4.239128 | 2.702480e-05 |
| 06.1. Expect-Home-Flood | -0.02804403 | 0.012849865 | 506.8396 | -2.182438 | 2.953555e-02 |
| 06.2. Time*Expect-Home-Flood | 0.07357018 | 0.017680869 | 412.5323 | 4.161005 | 3.857984e-05 |
| 07.1. Expect-Neighbourhood-Flood | 0.05203170 | 0.019022511 | 488.1668 | 2.735270 | 6.459771e-03 |
| 07.2. Time*Expect-Neighbourhood-Flood | -0.08963009 | 0.025588270 | 430.8828 | -3.502780 | 5.085551e-04 |
| 09.1. Dike-Protection | 0.12876297 | 0.035806887 | 599.2141 | 3.596039 | 3.497301e-04 |
| 10.1. Conscientiousness | 0.05849235 | 0.020784050 | 367.1806 | 2.814290 | 5.151785e-03 |
| 11.1. Openness | 0.04348044 | 0.017272592 | 372.4388 | 2.517309 | 1.224449e-02 |
| 12.1. Descriptive-Norms | 0.04378756 | 0.009872131 | 538.5800 | 4.435472 | 1.114297e-05 |
| 18.1. Male-Respondent | 0.15731591 | 0.038425480 | 378.3915 | 4.094052 | 5.185486e-05 |
| 19.1. N-Workers | 0.04893101 | 0.012423570 | 636.5184 | 3.938562 | 9.102684e-05 |
| 20.1. Membership-Local-Groups | 0.04152477 | 0.014219393 | 624.7464 | 2.920291 | 3.623140e-03 |
| **Intention model of retrofitting homes** |  |  |  |  |  |
| Predictor | Estimate | Std. Error | df | t value | Pr(>\|t\|) |
| (Intercept) | -0.54375669 | 0.13788645 | 655.7057 | -3.943511 | 8.894181e-05 |
| 00.1. Time | 0.28711764 | 0.08153678 | 537.4665 | 3.521327 | 4.658842e-04 |
| 06.1. Expect-Home-Flood | 0.04435207 | 0.01274857 | 648.7381 | 3.478983 | 5.370017e-04 |
| 08.1. Permanent-Home | -0.18381200 | 0.04906456 | 358.3354 | -3.746329 | 2.090472e-04 |
| 11.1. Openness | 0.04728118 | 0.01733924 | 353.3130 | 2.726830 | 6.713540e-03 |
| 14.1. Subjective-Norms | 0.05704062 | 0.02107871 | 684.1141 | 2.706078 | 6.977995e-03 |
| 15.1. Wishful-Thinking | 0.03705626 | 0.01689426 | 674.2735 | 2.193423 | 2.861761e-02 |
| 15.2. Time*Wishful-Thinking | -0.05837089 | 0.02221301 | 578.4629 | -2.627780 | 8.822493e-03 |
| 17.1. Age | -0.13733621 | 0.03903266 | 363.4198 | -3.518494 | 4.890599e-04 |
| **Behaviour model of retrofitting homes** |  |  |  |  |  |
| Predictor | Estimate | Std. Error | df | t value | Pr(>\|t\|) |
| (Intercept) | -0.33083388 | 0.062288699 | 398.4947 | -5.3112986 | 1.813679e-07 |
| 00.1. Time | -0.00713608 | 0.020047977 | 333.6678 | -0.3559501 | 7.221029e-01 |
| 02.1. Financial-Capacity | 0.01041520 | 0.004705813 | 341.6210 | 2.2132621 | 2.753998e-02 |
| 03.1. Worst-Flood-2022-2023 | -0.03338158 | 0.016470445 | 356.6359 | -2.0267564 | 4.343095e-02 |
| 04.1. Regular-Large-Flood | 0.14647567 | 0.051184722 | 372.8707 | 2.8617068 | 4.451276e-03 |
| 05.2. Time*Emotion-Effect | 0.01351183 | 0.005953322 | 336.2826 | 2.2696294 | 2.386280e-02 |
| 08.1. Permanent-Home | 0.23381877 | 0.062727621 | 371.5292 | 3.7275250 | 2.234740e-04 |
| 21.1. Moved-Permanent | 0.14073406 | 0.051781915 | 488.9317 | 2.7178227 | 6.804604e-03 |

Table H3. Detailed statistics of the coefficient differences of the same predictors on intention and behaviour

| **Predictor (Preparing devices)** | **Estimate** | **Std.Error** | **z.value** | **Pr(>\|z\|)** |
| --- | --- | --- | --- | --- |
| 00.1. Time | 0.50033913 | 0.25204590 | 1.9851112 | 6.340225e-01 |
| 01.1. Self-Efficacy | -0.05774058 | 0.02531921 | -2.2805045 | 3.855112e-01 |
| 02.1. Financial-Capacity | 0.09703184 | 0.02392444 | 4.0557616 | 1.128427e-03 |
| 02.2. Time*Financial-Capacity | 0.01858870 | 0.03294001 | 0.5643197 | 1.000000e+00 |
| 03.1. Worst-Flood-2022-2023 | 0.14575530 | 0.07624163 | 1.9117546 | 6.958152e-01 |
| 03.2. Time*Worst-Flood-2022-2023 | -0.34100695 | 0.10647677 | -3.2026419 | 3.099226e-02 |
| 04.1. Regular-Large-Flood | 0.44717866 | 0.06867371 | 6.5116428 | 1.353244e-09 |
| 04.2. Time*Regular-Large-Flood | -0.14755005 | 0.09490579 | -1.5547002 | 9.246268e-01 |
| 05.2. Time*Emotion-Effect | 0.06694237 | 0.02622422 | 2.5526926 | 2.088907e-01 |
| 06.1. Expect-Home-Flood | -0.09549643 | 0.02440272 | -3.9133518 | 2.126116e-03 |
| 06.2. Time*Expect-Home-Flood | 0.10773244 | 0.03706163 | 2.9068455 | 7.899174e-02 |
| 07.1. Expect-Neighbourhood-Flood | 0.11363732 | 0.03623391 | 3.1362150 | 3.830946e-02 |
| 07.2. Time*Expect-Neighbourhood-Flood | -0.18368798 | 0.05205270 | -3.5288848 | 9.541740e-03 |
| 09.1. Dike-Protection | 0.26036298 | 0.04816636 | 5.4054936 | 1.417535e-06 |
| 10.1. Conscientiousness | 0.06381843 | 0.02453651 | 2.6009577 | 1.849393e-01 |
| 11.1. Openness | 0.04408318 | 0.02807978 | 1.5699259 | 9.185404e-01 |
| 11.2. Time*Openness | -0.08425079 | 0.03995718 | -2.1085268 | 5.277212e-01 |
| 12.1. Descriptive-Norms | 0.04717789 | 0.02069744 | 2.2794071 | 3.867134e-01 |
| 13.1. Injunctive-Norms | -0.04262372 | 0.02384315 | -1.7876716 | 7.924089e-01 |
| 16.2. Time*Delaying | -0.05039503 | 0.03008476 | -1.6751016 | 8.660452e-01 |
| 17.1. Age | 0.16637001 | 0.04973549 | 3.3450967 | 1.846772e-02 |
| 18.1. Male-Respondent | 0.13617643 | 0.04670411 | 2.9157269 | 7.612768e-02 |
| 19.1. N-Workers | 0.05721491 | 0.02099713 | 2.7248916 | 1.327947e-01 |
| 20.1. Membership-Local-Groups | 0.01942975 | 0.02380253 | 0.8162896 | 9.999794e-01 |
|  |  |  |  |  |
| **Predictor (Retrofitting homes)** | **Estimate** | **Std.Error** | **z.value** | **Pr(>\|z\|)** |
| 00.1. Time | -0.219000021 | 0.13173367 | -1.6624453 | 6.980809e-01 |
| 02.1. Financial-Capacity | 0.041948549 | 0.01953575 | 2.1472715 | 3.223211e-01 |
| 03.1. Worst-Flood-2022-2023 | -0.131173691 | 0.05795434 | -2.2633970 | 2.514293e-01 |
| 04.1. Regular-Large-Flood | 0.133567975 | 0.05129289 | 2.6040251 | 1.075256e-01 |
| 05.2. Time*Emotion-Effect | 0.003182736 | 0.02829106 | 0.1124997 | 1.000000e+00 |
| 06.1. Expect-Home-Flood | -0.056523224 | 0.01715720 | -3.2944321 | 1.246241e-02 |
| 08.1. Permanent-Home | 0.385791706 | 0.06326911 | 6.0976318 | 1.195432e-08 |
| 11.1. Openness | -0.043604028 | 0.02175251 | -2.0045519 | 4.238934e-01 |
| 14.1. Subjective-Norms | -0.007929475 | 0.02823138 | -0.2808745 | 1.000000e+00 |
| 15.1. Wishful-Thinking | -0.007791667 | 0.02277171 | -0.3421643 | 9.999999e-01 |
| 15.2. Time*Wishful-Thinking | 0.046433236 | 0.03118162 | 1.4891218 | 8.215645e-01 |
| 17.1. Age | 0.152178977 | 0.04922140 | 3.0917237 | 2.462434e-02 |
| 21.1. Moved-Permanent | 0.033949257 | 0.05434582 | 0.6246894 | 9.998692e-01 |

**Appendix I. Boxplot Showing the Correlation Between Housing Situation and the Financial Capacity of Preparing Devices**


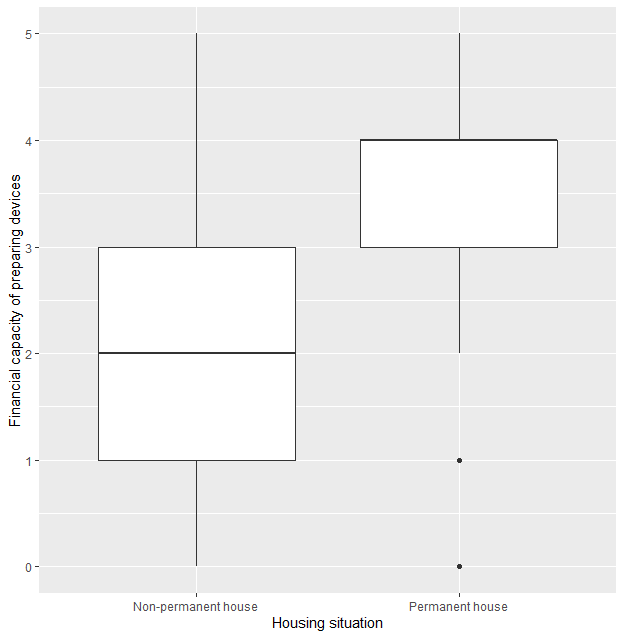


Figure I1. Financial capacity of preparing devices of respondents with different housing situations

The boxplot in Figure I1 shows that respondents with permanent houses have a higher perceived financial capacity for preparing devices than those with non-permanent houses. Furthermore, there is little overlap between the two boxes, indicating a strong correlation between the housing situation and the perceived financial capacity of preparing devices.

**References**

Bretz, F., Hothorn, T., & Westfall, P. (2010). *Multiple Comparisons Using R*. Taylor & Francis Group.

Cohen, J. (1992). A Power Primer, *112*(1).

Nicenboim, B., Schad, D., & Vasishth, S. (2024). *An Introduction to Bayesian Data Analysis for Cognitive Science.*

Norman, G. (2010). Likert scales, levels of measurement and the "laws" of statistics. *Advances in Health Sciences Education : Theory and Practice*, *15*(5), 625–632. https://doi.org/10.1007/s10459-010-9222-y

Vasishth, S., Daniel Schad, Audrey Bürki, & Reinhold Kliegl. (2023). *Linear Mixed Models in Linguistics and Psychology: A Comprehensive Introduction*. https://vasishth.github.io/Freq_CogSci/

West, B. T., Kathleen B. Welch, & Andrzej T. Gałecki. (2022). *Linear Mixed Models: A Practical Guide Using Statistical Software: Third Edition.*

1. *https://andrewpwheeler.com/2017/06/12/testing-the-equality-of-coefficients-same-independent-different-dependent-variables/; https://rpubs.com/bbolker/3336; https://stackoverflow.com/questions/78517026/multivariate-linear-mixed-model.* [↑](#footnote-ref-1)
